# AlloMAPS 2: allosteric fingerprints of the AlphaFold and Pfam-trRosetta predicted structures for engineering and design

**DOI:** 10.1093/nar/gkac828

**Published:** 2022-09-28

**Authors:** Zhen Wah Tan, Wei-Ven Tee, Enrico Guarnera, Igor N Berezovsky

**Affiliations:** Bioinformatics Institute (BII), Agency for Science, Technology and Research (A*STAR), 30 Biopolis Street, #07-01, Matrix, 138671, Singapore; Bioinformatics Institute (BII), Agency for Science, Technology and Research (A*STAR), 30 Biopolis Street, #07-01, Matrix, 138671, Singapore; Bioinformatics Institute (BII), Agency for Science, Technology and Research (A*STAR), 30 Biopolis Street, #07-01, Matrix, 138671, Singapore; Bioinformatics Institute (BII), Agency for Science, Technology and Research (A*STAR), 30 Biopolis Street, #07-01, Matrix, 138671, Singapore; Department of Biological Sciences (DBS), National University of Singapore (NUS), 8 Medical Drive, 117579, Singapore

## Abstract

AlloMAPS 2 is an update of the Allosteric Mutation Analysis and Polymorphism of Signalling database, which contains data on allosteric communication obtained for predicted structures in the AlphaFold database (AFDB) and trRosetta-predicted Pfam domains. The data update contains Allosteric Signalling Maps (ASMs) and Allosteric Probing Maps (APMs) quantifying allosteric effects of mutations and of small probe binding, respectively. To ensure quality of the ASMs and APMs, we performed careful and accurate selection of protein sets containing high-quality predicted structures in both databases for each organism/structure, and the data is available for browsing and download. The data for remaining structures are available for download and should be used at user's discretion and responsibility. We believe these massive data can facilitate both diagnostics and drug design within the precision medicine paradigm. Specifically, it can be instrumental in the analysis of allosteric effects of pathological and rescue mutations, providing starting points for fragment-based design of allosteric effectors. The exhaustive character of allosteric signalling and probing fingerprints will be also useful in future developments of corresponding machine learning applications. The database is freely available at: http://allomaps.bii.a-star.edu.sg.

## Graphical Abstract

**Graphical Abstract:**
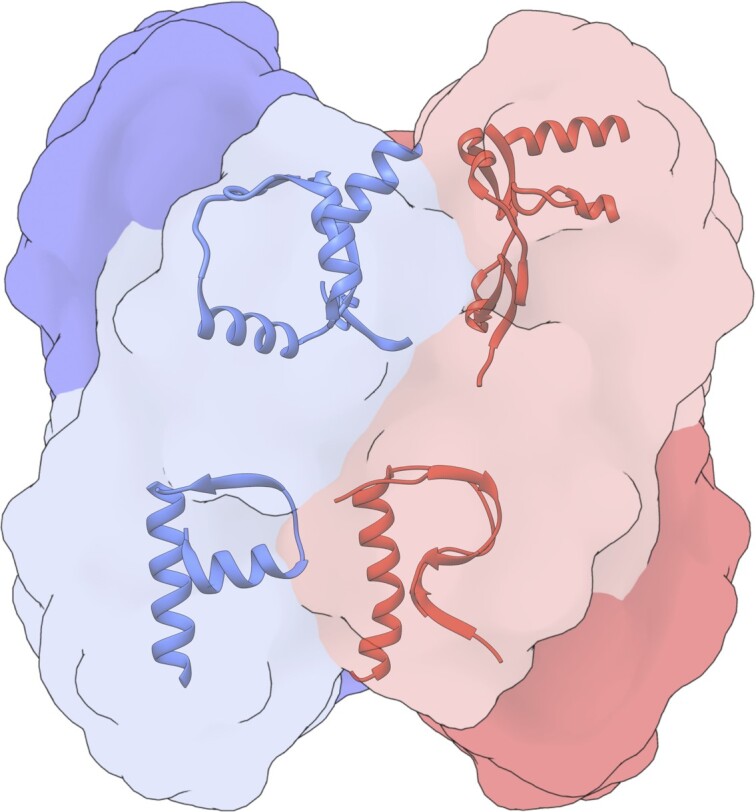
AlloMAPS update: allosteric fingerprints of proteins in AlphaFold database and trRosetta-predicted Pfam domains.

## INTRODUCTION

Despite constantly growing interest in biomedical implications of allostery in general (1,2) and in design of allosteric effectors in particular (3–6), there is only a handful number of clinically approved allosteric drugs most of which were discovered serendipitously (4). Moreover, it is increasingly recognized that well-established principles and protocols in the screening of ligand libraries against traditional drug targets and their binding sites are not optimal for the identification and design of allosterically acting medicines (7). The quest for allosteric drugs should be based, therefore, on completely different principles determined by distinct characteristics of allosteric sites and effectors (7,8) and by the structural dynamics-based perturbative nature of allostery (9,10). Specifically, it was suggested that this procedure should start from the search of latent (11) or design of new non-natural allosteric sites (12) followed by mutual design and adjustments of the allosteric ligand-site pair in order to achieve required effects (7). The proposed framework is based on the Structure-Based Statistical Mechanical Model of Allostery (SBSMMA, (9,13)), which provides quantification of allosteric signalling with per-residue resolution, allowing, thus, to tune ligand-site interactions in order to obtain desired allosteric effects (14). Acknowledging the omnipresence of allostery (6) and advantages of prospected allosteric drugs (4–6), which are supported by recent progress in quantitative description of allostery, one may conclude that practically any disease-causing protein can be considered as a target for allosteric drug development (4,7). Recent progress in AI-based structure predictions (15,16) delivered the structures of a large number of potential yet unexplored drug targets with uncharacterized allosteric signalling. The goal of this update is to provide a comprehensive information on allosteric communication in predicted structures from the AlphaFold (17) and Pfam (18) databases. In addition to Allosteric Signalling Maps (ASMs), which exhaustively describe the signalling in per-residue approximation and allosteric effects of mutations, we computed the Allosteric Probing Maps (APMs) quantifying the allosteric effects of small probe binding, which can be used in fragment-based design of allosteric effectors (7,14,19). We provide corresponding information for 12 model organism proteomes and 14 proteomes of various pathogens involved in global health issues presented in AlphaFold protein structure database (17) and for the set of trRosetta-predicted (15) Pfam domains (18).

## THEORETICAL BACKGROUND AND COMPUTATIONAL METHODS

**Figure 1.**
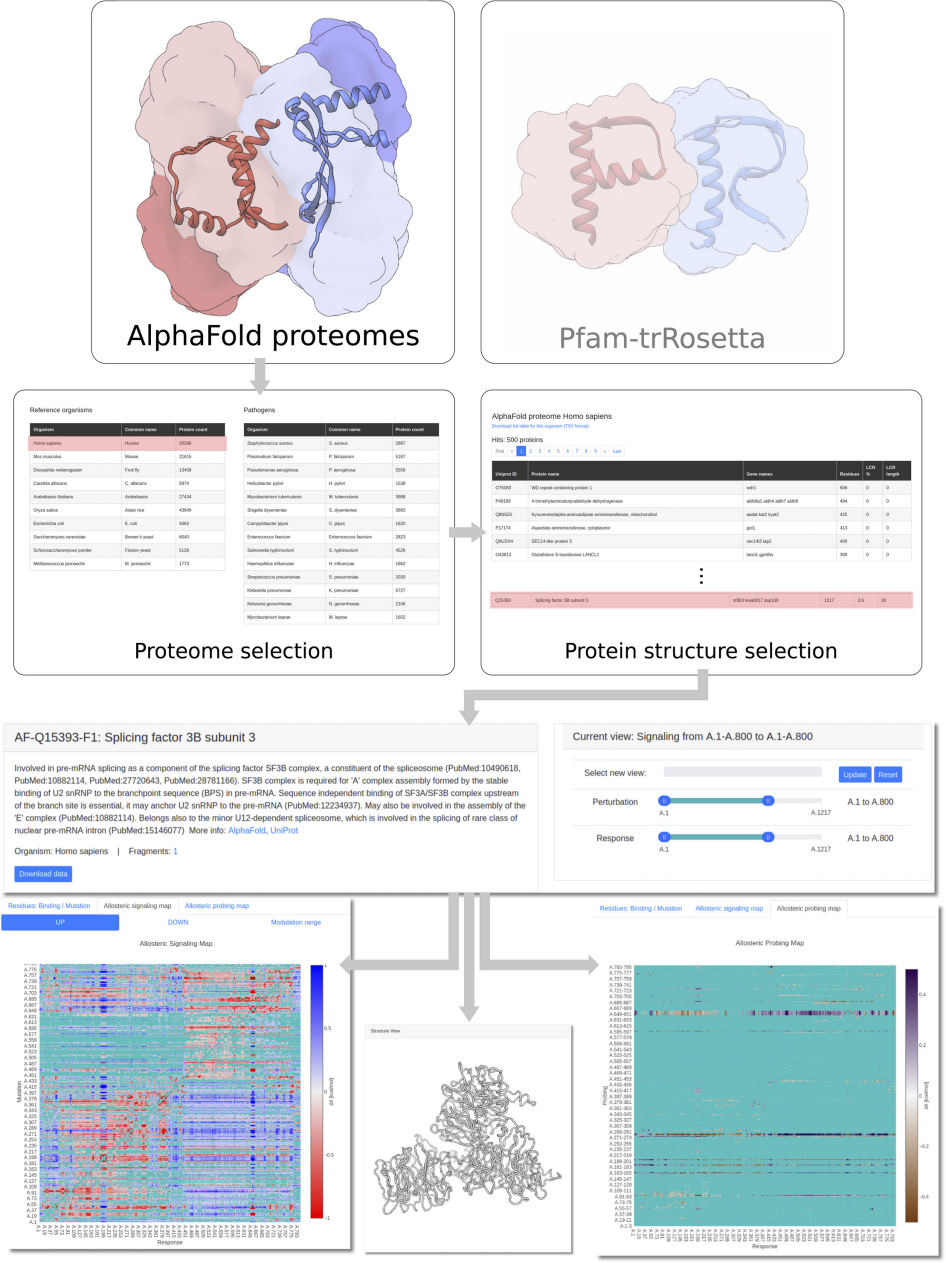
Flowchart of the datasets added to the AlloMAPS 2 database and its updated features. First row: selection of the dataset, AlphaFold or Pfam-trRosetta. Second row: selection of the organism (left panel) in AlphaFold dataset and of the protein (in both datasets). Third row: description of the protein (left) and optional selection of parts of ASM/APM in case of large structures with more than 1200 residues. Bottom row: selected part of ASM/APM (left/right) and structure (center) of considered here Splicing factor 3B subunit 3.

We quantified allosteric communication on the predicted structures using the benchmarked Structure-Based Statistical Mechanical Model of Allostery (SBSMMA, (9)). The model outputs are presented in form of allosteric signalling/probing maps (ASM/APMs, (13,19)), which show changes in per-residue free energy in response to signals caused by a perturbation: single-residue mutations or small-probe binding to three-residue segments in case of ASM and APM, respectively. The per-residue free energy difference (kcal/mol) between the native $( 0 )$ and perturbed $( P )$ states is estimated for each residue $i$, $\Delta \ g_i^{( P )} = \frac{1}{2}\ {k}_BT\mathop \sum \limits_\mu \ln \frac{{\varepsilon _{\mu ,i}^{( P )}}}{{\varepsilon _{\mu ,i}^{( 0 )}}},$ which depends exclusively on the parameters $\varepsilon _{\mu ,i}$ that characterize the native $( 0 )$ and perturbed $( P )$ protein conformational ensembles. Briefly, the free energy ${g}_i = \ - {k}_BT\ln {z}_i$ is obtained from the per-residue partition function ${z}_i = \mathop \prod \limits_\mu {( {\pi 2{k}_BT/{\varepsilon }_{\mu ,i}} )}^{1/2}$, which, in turn, is a result of the integration of the allosteric potential over all possible displacements of neighbors of the residue $i$. The allosteric potential ${U}_i\ ( \sigma ) = \ 1/2\mathop \sum \limits_\mu {\varepsilon }_{\mu ,i}\sigma _\mu ^2$ evaluates the effect of a perturbation on a residue *i* as the elastic work (the implementation of SBSMMA used here is based on a Cα harmonic model of proteins) exerted on *i* due to changes in the displacements of surrounding residues *j* caused by normal modes ${{\boldsymbol{e}}}_\mu$, where ${\varepsilon }_{\mu ,i} = \mathop \sum \limits_j {| {{{\boldsymbol{e}}}_{\mu ,i} - {{\boldsymbol{e}}}_{\mu ,j}} |}^2$ and $\sigma \ = ( {{\sigma }_1, \ldots ,\ {\sigma }_\mu ,\ \ldots } )$ is a set of Gaussian distributed amplitudes with variance 1/${\varepsilon }_{\mu ,i}$ (see for details (10,13,14)). We evaluate the allosteric modulation as a deviation of the obtained free energy difference from its mean value over the protein chain, eliminating thus the background allosteric signalling: ${\rm{\Delta }}h_i^{( P )} = \ {\rm{\Delta }}g_i^{( P )} - {\langle {\rm{\Delta }}g_i^{( P )}\rangle }_{Chain/Protein}$. Allosteric modulation close to zero indicates that signalling to the residue/site of interest is similar to the background effect on the whole chain/protein. In terms of mutations, we modelled the effect of UP ($m \uparrow$) mutations, which represent a substitution of residue $m$ to the bulkiest ones (for example Phe or Trp), and the resultant free energy change at responding residue $i$ is expressed as ${\rm{\Delta }}h_i^{( {m \uparrow } )}$. Similarly, DOWN ($m \downarrow$) mutations model the opposite conversion of a residue $m$ to the smallest amino acid (Gly-like), and the effect on residue $i$ is expressed as ${\rm{\Delta }}h_i^{( {m \downarrow } )}$. The allosteric modulation range (kcal/mol) on responding residue $i$${\rm{\Delta \ }}h_i^{( {m \downarrow \uparrow } )} = \ {\rm{\Delta }}h_i^{( {m \uparrow } )} - {\rm{\Delta }}h_i^{( {m \downarrow } )}$ is a measure of the overall allosteric effect on residue $i$ upon a substitution of the smallest amino acid by the largest one at residue position $m$, providing a generic description of the maximal signalling strength from position $m$ to $i$. For amino acid changes in the opposite direction, namely from the largest to the smallest residues, the modulation range is $\Delta \ h_i^{( {m \uparrow \downarrow } )} = \ - \Delta h_i^{( {m \downarrow \uparrow } )}$. Since the signal obtained upon conversion between the largest and the smallest amino acids is the same in absolute value regardless of the direction of the substitution, it serves as an indicator of the dynamic range of the allosteric signal that can be obtained upon mutations of a considered position in the protein sequence/structure. Exhaustive quantification of signalling for all pairwise residue positions is represented in the ASM matrix plot (Figure 1). The ASM reveals distant residues/protein regions that are allosterically connected with strong positive or negative signalling, which might indicate conformational changes or stabilization in the affected residues/regions, respectively. On the other hand, to understand and explore the effect of ligand binding to different parts of a protein chain, we exhaustively simulate the binding of a small probe to every consecutive three-residue segment along a protein chain (see (14) for technical details). The effect of a probe binding on every residue $i$, ${\rm{\Delta }}h_i^{( {Probe} )},$ is represented in the APM matrix plot (Figure 1, for details of APM calculation see (14)). The ASM/APM matrices are presented here in the form of allosteric fingerprints, in which the signalling between proximal residues (see (12,20) for explanations of proximity according to an operational definition of allosteric distances) and/or those with negligible modulation are color-coded (in blue-green), allowing the user to focus on the relevant and strong allosteric communication.

## DESCRIPTION OF THE DATABASE UPDATE

The original database (21) contained data on allosteric signalling in more than 2000 proteins and protein chains grouped in three sets: (i) ‘Allosteric proteins’ with the data on allosteric regulation in 48 proteins documented on the basis of experimental works (9,12) originally published in (22); (ii) ‘PDBselect chains’ set of 1908 protein chains contains representative structures with low sequence identity (less than 25%) providing a ‘list of representative protein chains with low mutual sequence identity selected from the protein data bank (PDB, (23)), which enables unbiased statistics’ (24); (iii) ‘Allosteric polymorphism’ set with 33 proteins (21) with multiple (more than 50 in each protein) known pathology-related SNPs originally obtained in (25) based on the UniprotKB/Swiss-Prot (26) Humsavar database (version 2017_09; current version is available at: https://ftp.uniprot.org/pub/databases/uniprot/current_release/knowledgebase/complete/docs/humsavar) containing all missense variants annotated in human entries.

The current update includes high-throughput data on allosteric signalling and effects of probing in the predicted structures from the AlphaFold (16) protein structure database (17) and the trRosetta (15) predicted set of Pfam (18) domains. The original collections of structures were filtered in order to select only structures with sizes larger than typical size of protein domains/folds (27,28) and with sufficient quality of predictions (see Materials and Methods for the description of filtering procedure). Table 1 contains a list of organisms presented in the AlphaFold database with the total number of initial protein entries available for download and the number of high-quality structures available for browsing in AlloMAPS 2. Remaining structures not satisfying the filtering criteria are available through the search using their accession code listed in downloadable complete lists of proteomes (see online Tutorial and Materials and Methods for details).

**Table 1.** List of AlphaFold proteomes included in the AlloMAPS 2 database update. Quality filters were applied to the predicted structures, requiring that low-confidence residues (LCRs) constitute only a small fraction of the structure and do not form long consecutive segments. Structures with low-confidence signal peptides were also excluded. The Browse function for the database focuses on reasonably large structures with more than 100 residues, yielding about 50 000 proteins in all proteomes presented in this table

Organism name	AlphaFold entries with full structure	Selected entries for browsing
***Homo sapiens***	20 296	4073
***Mus musculus***	21 615	4989
***Drosophila melanogaster***	13 458	2823
***Danio rerio***	24 664	5032
***Candida albicans***	5974	1598
***Caenorhabditis elegans***	19 694	4866
***Arabidopsis thaliana***	27 434	5126
***Oryza sativa***	43 649	5221
***Escherichia coli***	4363	2979
***Saccharomyces cerevisiae***	6040	1540
***Schizosaccharomyces pombe***	5128	1515
***Methanococcus jannaschii***	1773	1305
***Staphylococcus aureus***	2887	1874
***Plasmodium falciparum***	5187	683
***Pseudomonas aeruginosa***	5556	3832
***Helicobacter pylori***	1538	939
***Mycobacterium tuberculosis***	3988	2347
***Shigella dysentriae***	3893	2469
***Campylobacter jejuni***	1620	1217
***Enterococcus faecium***	2823	2504
***Salmonella typhimurium***	4526	3101
***Haemophilus pneumoniae***	1662	1180
***Streptococcus pneumoniae***	2030	1177
***Kiebsiella pneumoniae***	5727	3784
***Neisseria gonorrhoeae***	2106	1246
***Mycobacterium leprae***	1602	875

## MATERIALS AND METHODS

The predicted structures of 12 key model organisms and 14 pathogens from the AlphaFold protein structure database (https://alphafold.ebi.ac.uk/download, (17)) and of 6370 protein families from the Pfam database (http://ftp.ebi.ac.uk/pub/databases/Pfam/releases/Pfam33.1/structure_models/, (18)) were filtered in order to focus on high-quality structures contained in these large datasets. Confidence of residue-level structures were estimated in AlphaFold (17) using a measure called pLDDT, which is based on the lDDT (local Distance Difference Test) metric (29) obtained by comparing pairwise distances of C-alpha atoms between predicted and existing reference structures. First, in both AlphaFold and trRosetta predictions, a residue having pLDDT score lower than 70 (equivalently, lDDT score lower than 0.7) indicates low confidence in the prediction. Hence, a structure prediction of high quality should have few or no low-confidence residues (LCRs), and should not have long continuous stretches of low-confidence residues. In alignment with the original AlphaFold publication (17), residues with scores pLDDT <70 are considered low-confidence residues (LCRs). To this end, we required that the percentage of LCRs in each structure (listed under the column lcr_perc in the downloadable table of structures) be lower than a given threshold (10% for prokaryotes and 20% for eukaryotes), and that the longest continuous stretch of LCRs (lcr_length) be shorter than a given threshold (15 residues for prokaryotes, 25 for eukaryotes)—we set this limit on the basis of polymer nature of protein backbone determining the size of protein chain returns known as closed loops (30,31). We adopted looser requirements for eukaryotes due to the presence of relatively larger unstructured or disordered regions in eukaryotic proteins (27). To exclude structures containing low-confidence signal peptides, we further required that the first 20 residues from the N-terminus are not all LCRs. Finally, we considered only structures that are larger than 50 residues in size, guaranteeing at least the protein fold size for considered structures (27,28). Corresponding numbers of proteins remaining after selection according to above criteria are listed in Supplementary Table S1. The Browse function shows only structures with >100 residues, yielding more than 50 000 high-quality structures (see Table 1) with sizes exceeding the minimal size of protein domain/fold (28). Other structures can be accessed through the search function using their accession code listed in the downloadable complete lists of proteomes (see online Tutorial for details).

## ORGANIZATION OF THE NEW DATASETS, THEIR NAVIGATION AND USAGE

Figure 1 shows a flowchart navigating through the updated parts of the database, as well as generically implemented new features. To browse AlphaFold structures, users can first select a proteome of interest. Clicking on the AlphaFold panel (shown in Figure 1 along with the Pfam-trRosetta starting panel) in the AlloMAPS home page brings users to the list of organisms with AlphaFold proteomes available in the database. Selecting an organism of interest (Figure 1, second row, left panel) generates a list of predicted structures satisfying our quality control criteria and passing the filtering procedure (see Materials and Methods), which enables the users to identify a structure of interest. The list of proteins (Figure 1, second row, right panel) starts from structures with the smallest proportion of low-confidence residues. Examples of top25 entries in each organism are presented in Supplementary Figure S1. Selecting a given structure brings the user to the interactive data dashboard schematically shown in the third level (left panel), where further information about the protein is provided, with links to relevant external resources (for example, AlphaFold and Uniprot).

Accessing the structure of interest, the users can study how structural perturbations in different parts of the protein can affect distant regions allosterically. In the original version of AlloMAPS, users were able to study the effect of individual mutations by selecting these residues on the ‘Residues: Binding/Mutation’ panel, and also visualize the allosteric signalling map (ASM) as a matrix plot in the corresponding panel. These features have been retained, and we have also added the pre-computed allosteric probing map (APM) for each structure in this update. Furthermore, we have updated the matrix view of the allosteric signalling/probing maps using allosteric fingerprints, masking the effects due to proximal regions and/or weak signalling. Large structures (above 1200 residues) may be studied by zooming into selected, smaller regions of interest (Figure 1, third row, right panel). In case of using the Pfam-trRosetta dataset, the user starts from the corresponding panel on the AlloMAPS homepage, which will open the page with a list of structures, similar to the one for AlphaFold proteins (second row, right panel). Some tasks may require additional and/or preliminary analysis: as an example, oligomerization will definitely originate additional signalling, which will be taking place between protomers and may affect to some extent the original signalling in individual protomers. Therefore, while signalling in protomers can be obtained from the presented database, in order to analyze a complete picture of allosteric signalling in oligomers, it will be necessary to obtain accurate structures of oligomers as an input for the AlloSigMA server (19) to compute ASMs/APMs. Noteworthy, some low-quality AlphaFold predictions may require special treatments. For example, in case of multi-chain and/or oligomeric proteins the AlphaFold predictions should be performed first for individual chains/protomers followed by the assembly into the final structure.

Figure 1 illustrates the example of splicing factor 3B subunit 3, consisting of 1217 amino acid residues, of which first 800 residues were selected (third row, right panel) for building the ASM and APM fingerprints. We would like to specifically emphasize on the appearance of the APM fingerprint. The APM fingerprint (bottom row, right panel) contains, unlike the corresponding ASM’s fingerprint (bottom row, left panel), more areas featuring negligible signalling strength and at short distances, which are filtered out from the original APM (these areas are blue-green masked in the fingerprint). Nevertheless, it is a meaningful result indicating that only a limited number of places can be targeted in order to obtain a strong allosteric signal originated by the probe binding. Another technical improvement we have implemented in this update is the ability to visualize large structures that were previously inaccessible due to memory constraints: for all structures above 1200 residues in size, an extra panel is included to enable users to zoom in on different, smaller regions to examine signalling data on applied perturbation and responses.

## CONCLUSIONS

Constantly improving resolution of experimental techniques, such as X-ray crystallography, NMR, and cryoEM and their transformation into high-throughput approaches have provided wealth of experimental data, on protein structures and sequences, in particular. It has allowed, in turn, to use the power of machine learning for predicting protein structures that are difficult to obtain via experiments (15,16). Advantages of allosteric medicines (4–7) over traditional orthosteric ones call for their development targeting established and new drug targets. At the same time, observations on mutations (4,25) triggering different pathologies via allosteric signalling point to the need of their characterization and consideration in diagnostics (6). The significantly expanded array of potential drug targets provided by the AI-based predictions of protein structures (17,18) is especially important for answering the quests for precision/personalized medicine, motivating us to perform this update of the AlloMAPS database. We provide here about 50 000 fingerprints of Allosteric Signalling and Probing Maps (ASMs/APMs) for newly predicted high-quality structures. This update allows to expand the analysis of allosteric communication from the effects of ligand binding and mutations to engineering and design of allosteric signalling (20) in newly predicted structures, as well as to target them with newly developed allosteric effectors (7). Specifically, the ASM fingerprints will facilitate diagnostics of different pathologies, helping to predict allosteric effects of mutations and their combinations, and analyzing their role in expansion of cancer landscapes (32) and triggering other diseases (5,6). One of the recent examples of using ASMs as a diagnostic tool demonstrated successful prediction of distant mutations (many of which are associated with variants of concern including the Omicron variant) that might affect the dynamics of the receptor-binding domain of the SARS-CoV-2 Spike protein (33). The APM fingerprints will be useful in fragment-based design of allosteric drug candidates. The comprehensive nature of ASM/APM fingerprints available in the AlloMAPS database along with extensivity of free energy accounting for allosteric effects in SBSMMA allows one to use these data as an input for future machine learning approaches (34,35) on diagnostics and drug design (36).

The outlook on future work presents several promising research directions that can be explored with the SBSMMA-based (9,12–14) computational suite comprised of the AlloSigMA (19,37) web-server and the AlloMAPS database, as well as corresponding requirements for their further developments. The high-throughput data presented in this update provides an overall picture of allosteric signalling in predicted structures of proteins important in biomedical and biotechnological applications, which can serve as a starting point in diagnostics and engineering/design efforts involving these proteins. Further steps in these efforts may require more accurate consideration of allosteric effects, which can be achieved within the SBSMMA framework by replacing the input normal modes obtained from a C_α_ harmonic model of the protein backbone with the principal components of the covariance matrix obtained in atomistic MD simulations of corresponding structures (4,7). The MD-based approach comes, however, at the expense of increased computing time, making it necessary, for practical purposes, to combine data obtained in atomistic simulations with the massive coarse-grained one presented in AlloMAPS. The high-throughput data would also be instrumental for validation and calibration of computational predictions in conjunction with experimental assays (2,7), such as deep mutational scanning and fragment-based screening. The capability of artificial intelligence (AI) algorithms (34–36) in performing feature extraction from low-level data representation with non-obvious hierarchical connectivity and to model complex nonlinear input-output relationships can be utilized on the basis of these data. All the above will provide an opportunity to include allosteric regulation as an integral part of protein design procedures (38–41) and to improve protocols for design of allosteric drugs (4,5,7), which, in turn, will allow to tackle challenging tasks of targeting so-called undruggable targets (42,43) and regulating multiscale cellular signalling.

## DATA AVAILABILITY

The database is freely available at: http://allomaps.bii.a-star.edu.sg.

## Supplementary Material

gkac828_Supplemental_Files

## Notes

Present address: Enrico Guarnera, Global Analytical Pharmaceutical Science and Innovation, Merck KGaA, Via Luigi Einaudi, 11, Guidonia Montecelio, 00012 Rome, Italy.
