# Supplementary material for: AlloMAPS 2: allosteric fingerprints of the AlphaFold and Pfam-trRosetta predicted structures for engineering and design
Source: Nucleic Acids Res. 2022 Sep 28;51(D1):D345–51. doi: 10.1093/nar/gkac828 (PMC9825619; doi:10.1093/nar/gkac828)
Supplement: gkac828_Supplemental_Files [file gkac828_supplemental_files.zip › SI_Titles.docx]

**Supplementary Table 1.** Numbers of proteins in AlphaFold and trRosetta-Pfam predicted sets sequentially selected in steps of filtering procedure.

**Supplementary Figure 1.** Examples of top 25 entities selected in each organism of the AlphaFold database via filtering procedure.
