# Supplementary material for: AlloMAPS 2: allosteric fingerprints of the AlphaFold and Pfam-trRosetta predicted structures for engineering and design
Source: Nucleic Acids Res. 2022 Sep 28;51(D1):D345–51. doi: 10.1093/nar/gkac828 (PMC9825619; doi:10.1093/nar/gkac828)

HUMAN catalog top 25 entries

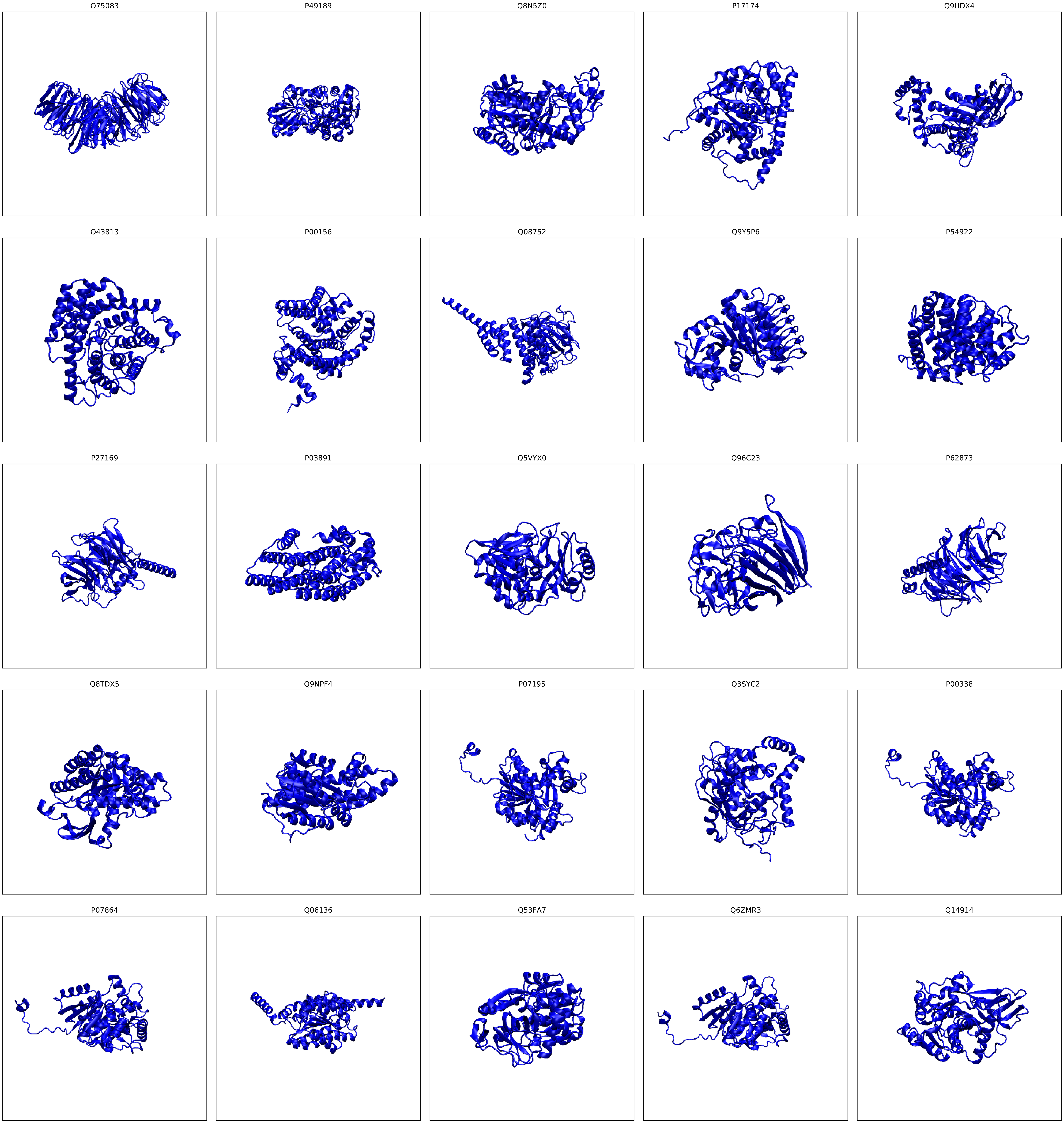

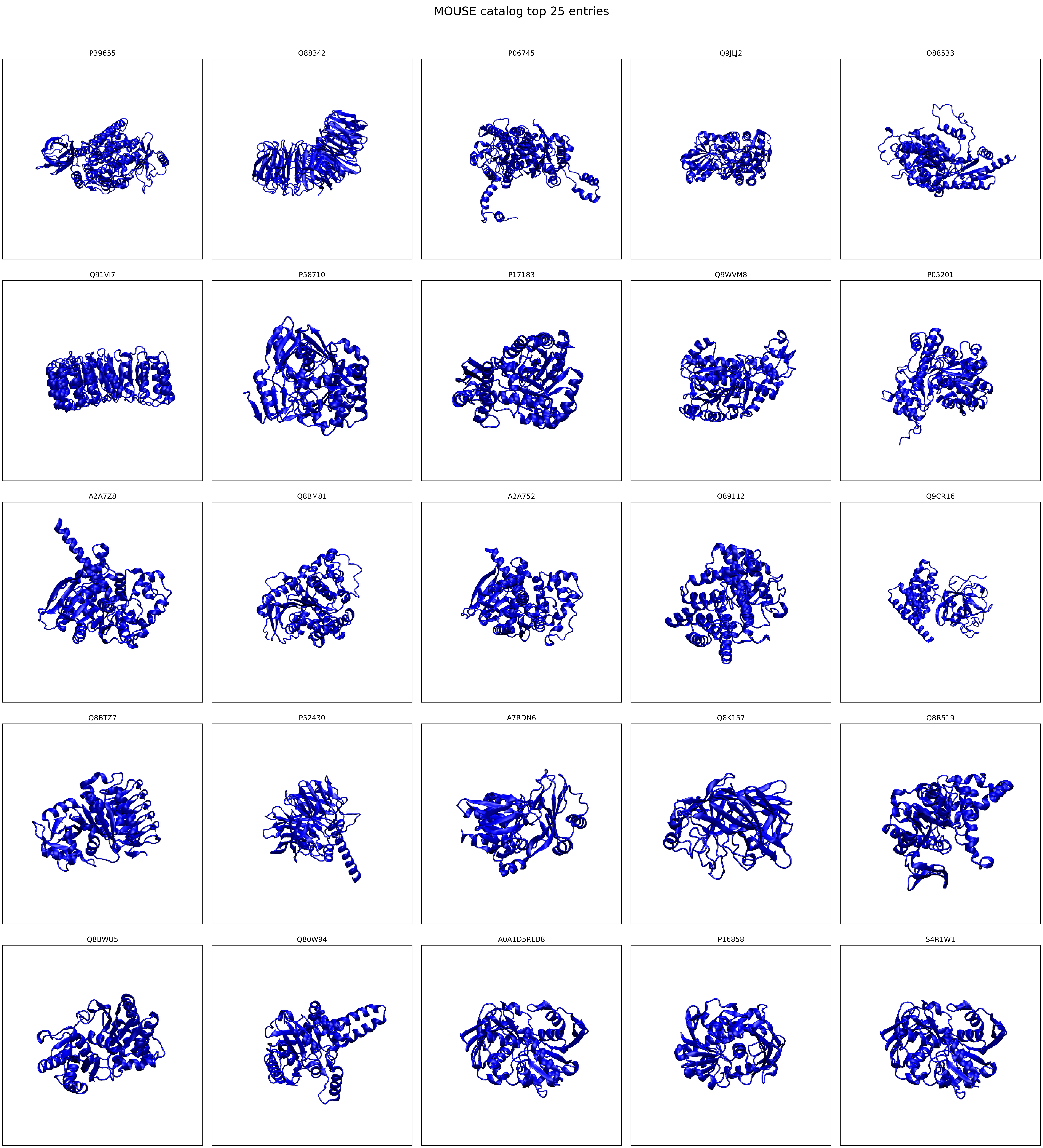
MOUSE catalog top 25 entries
P39655
O88342
P06745
Q9JLJ2
O88533
Q91VI7
P58710
P17183
Q9WVM8
P05201
A2A7Z8
Q8BM81
A2A752
O89112
Q9CR16
Q8BTZ7
P52430
A7RDN6
Q8K157
Q8R519
Q8BWU5
Q80W94
A0A1D5RLD8
P16858
S4R1W1

DROME catalog top 25 entries

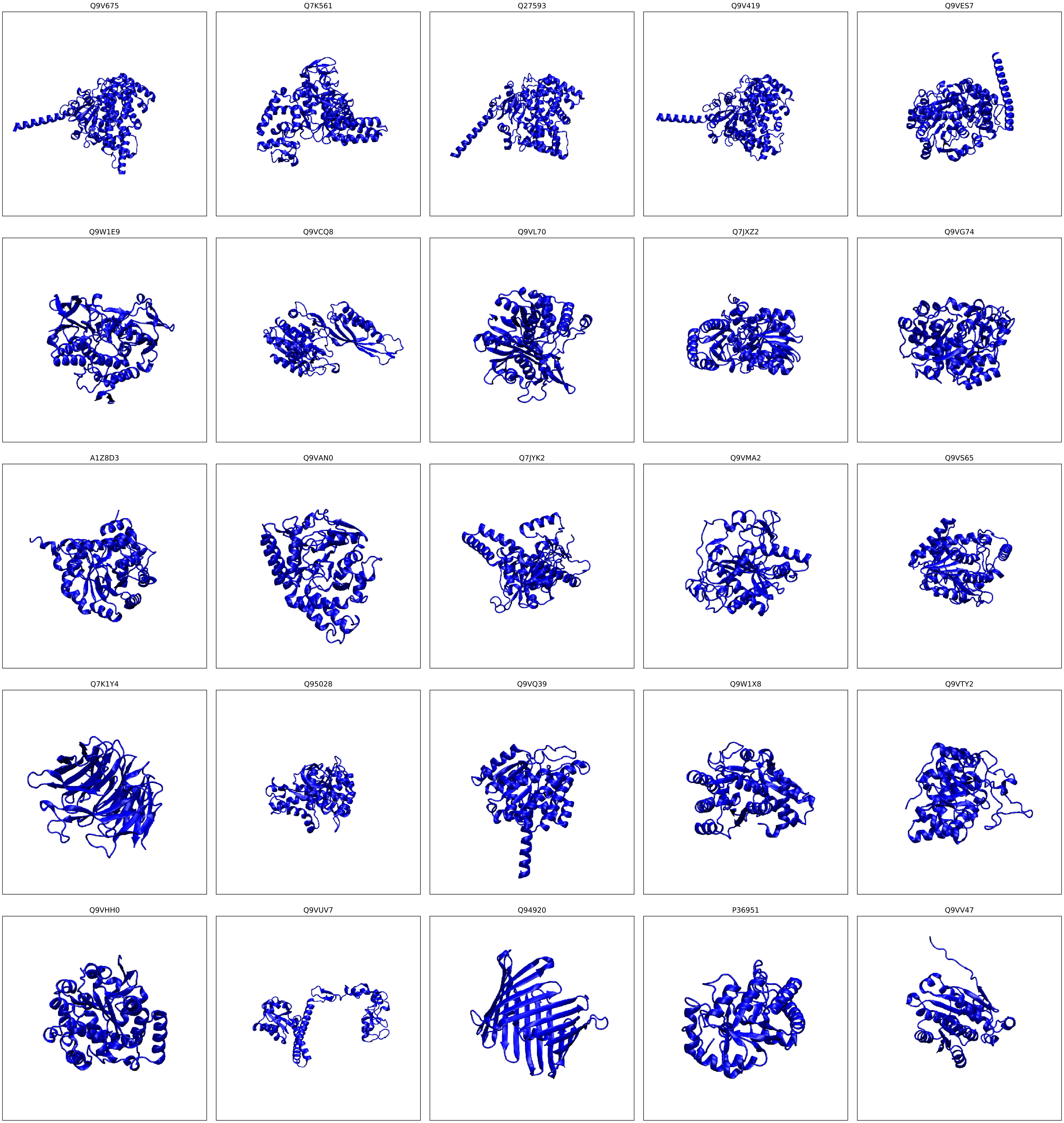

CAEEL catalog top 25 entries

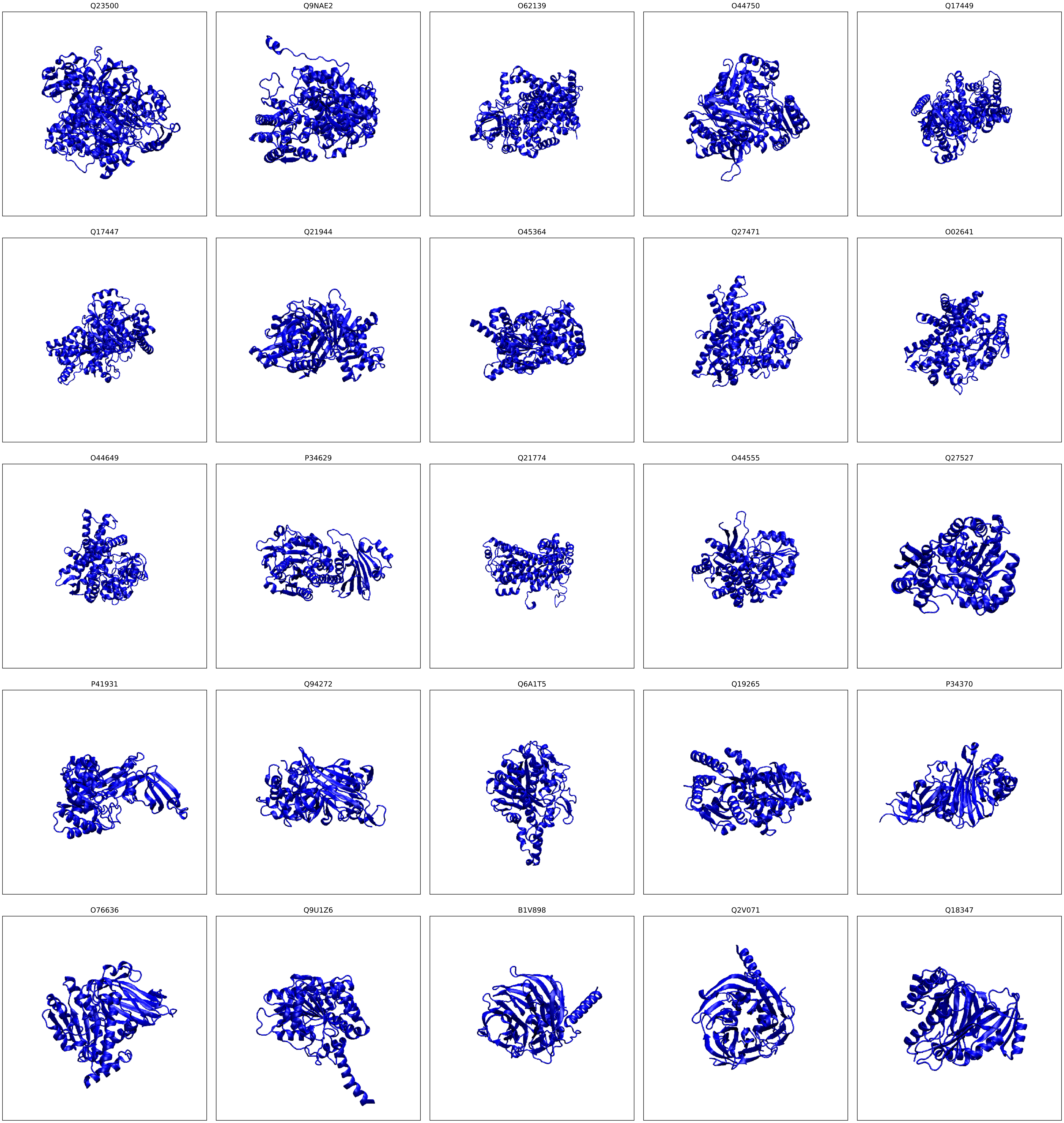

DANRE catalog top 25 entries

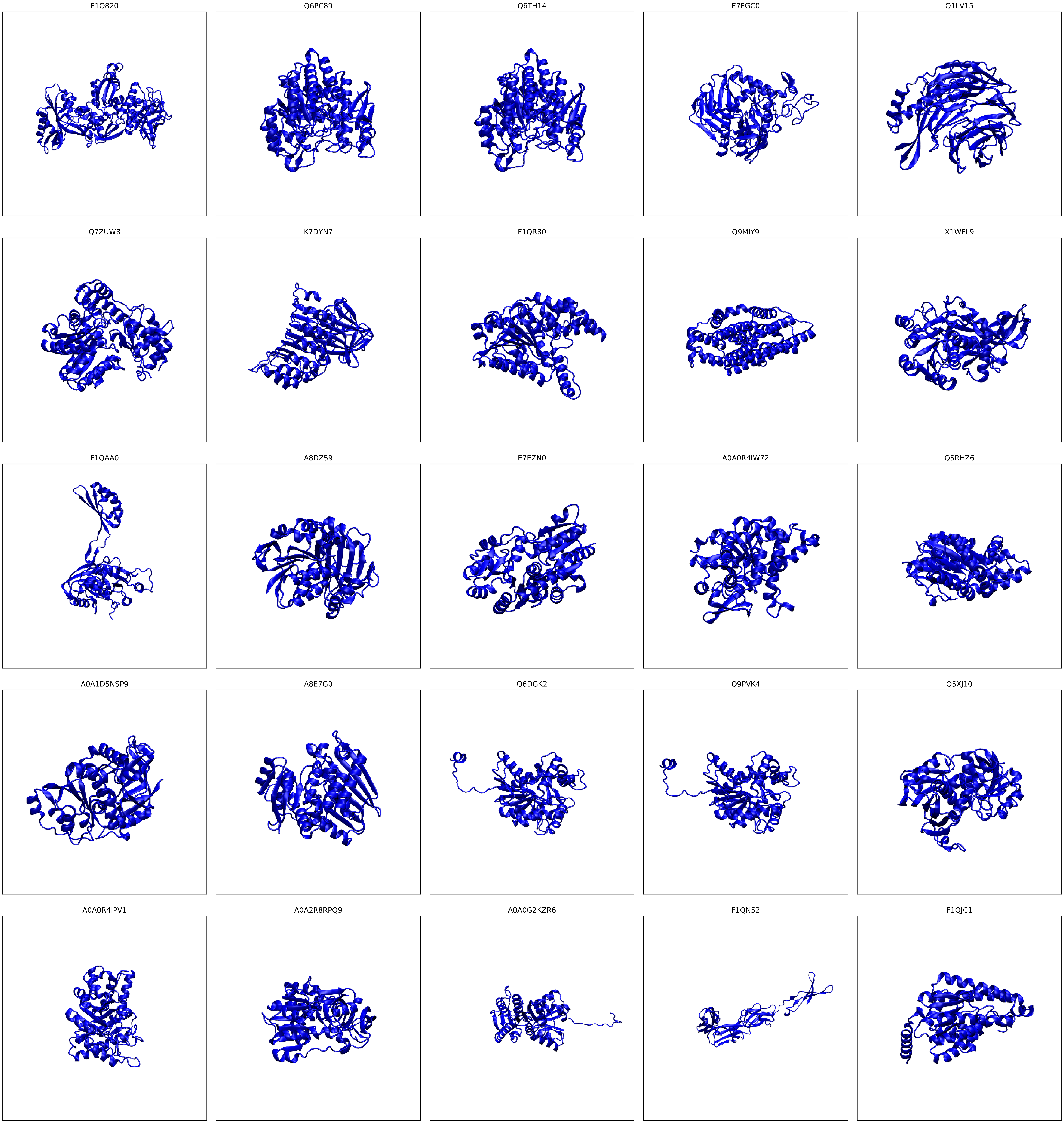

CANAL catalog top 25 entries

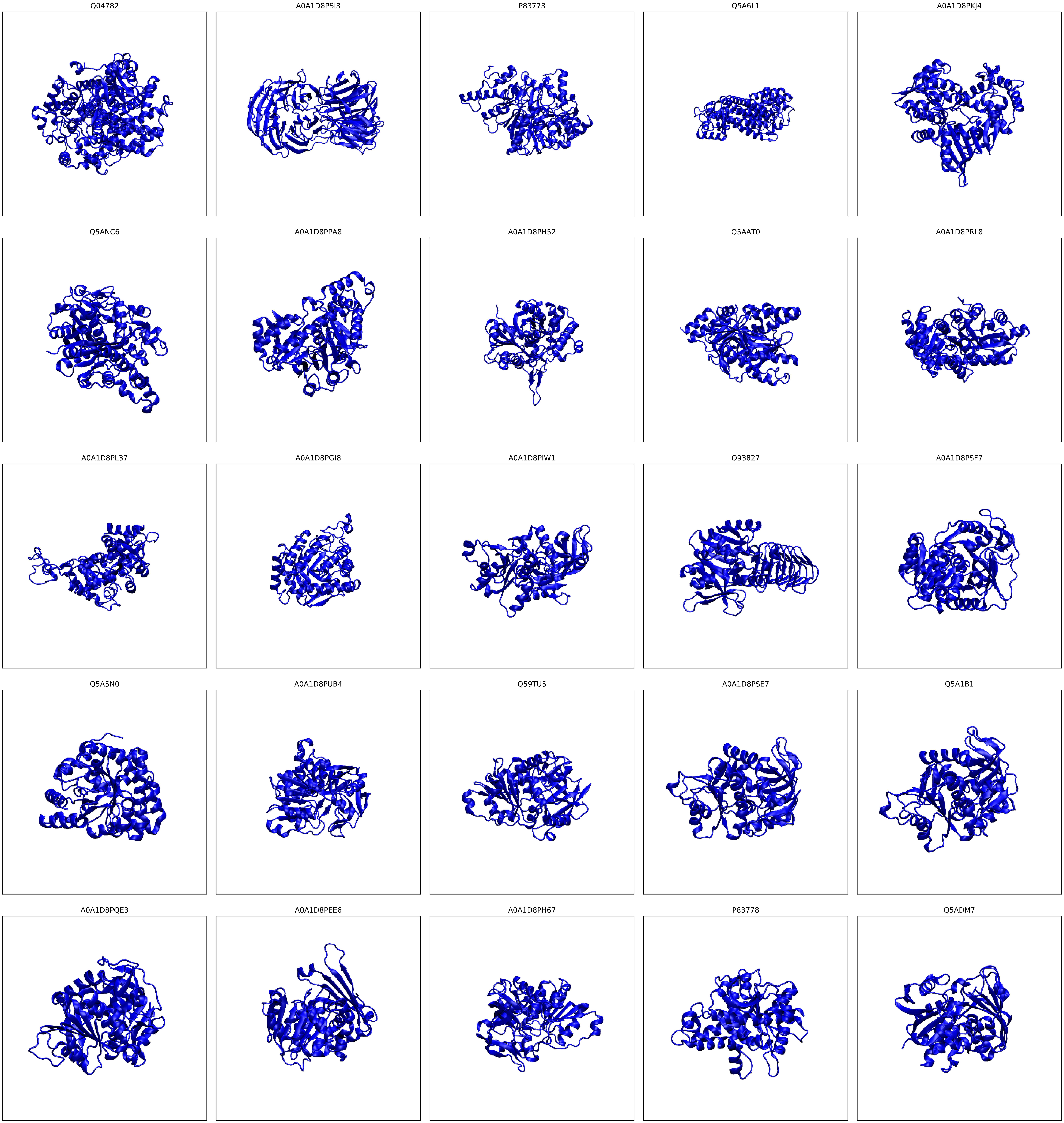

ARATH catalog top 25 entries

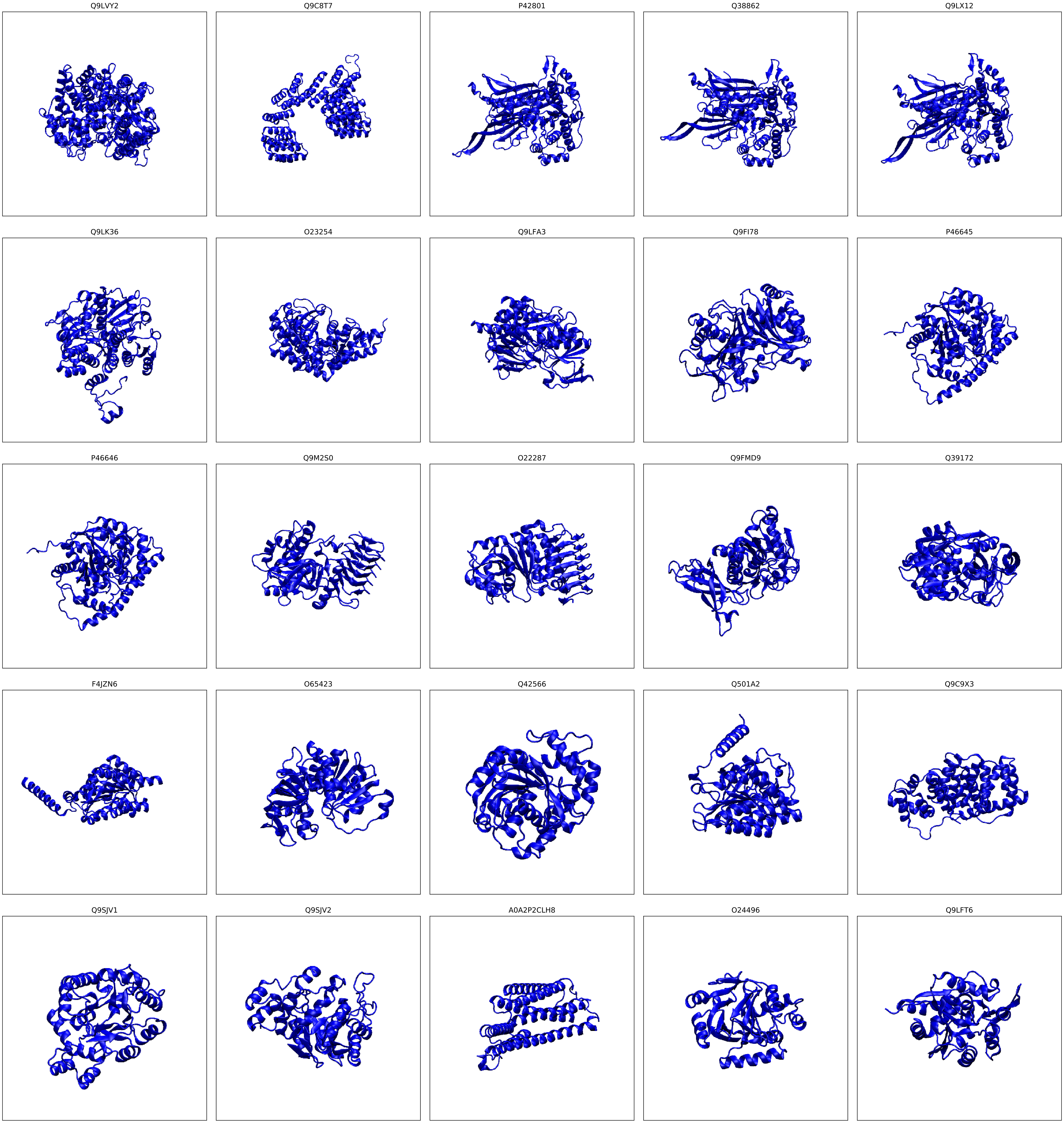

ORYSJ catalog top 25 entries

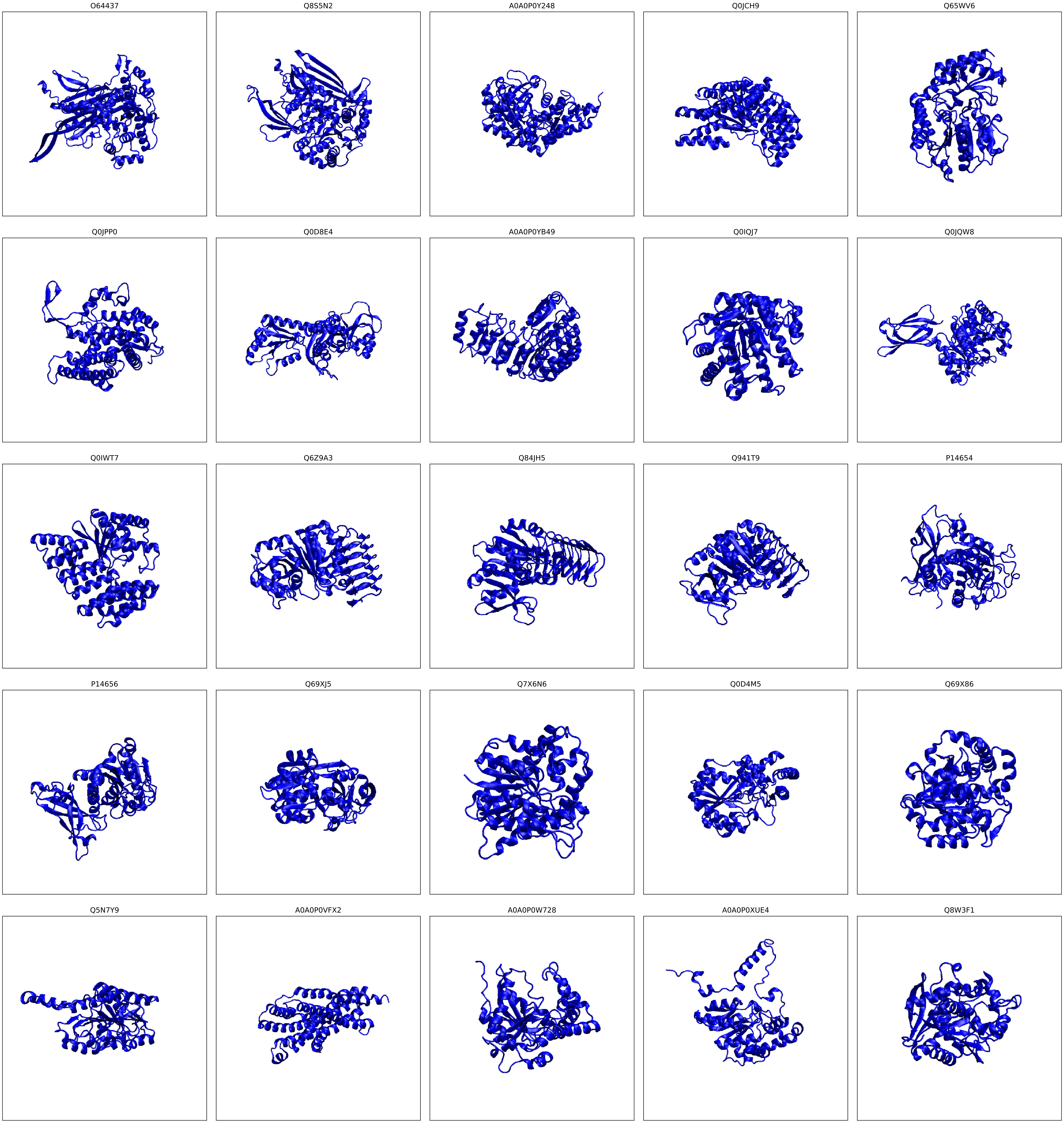

ECOLI catalog top 25 entries

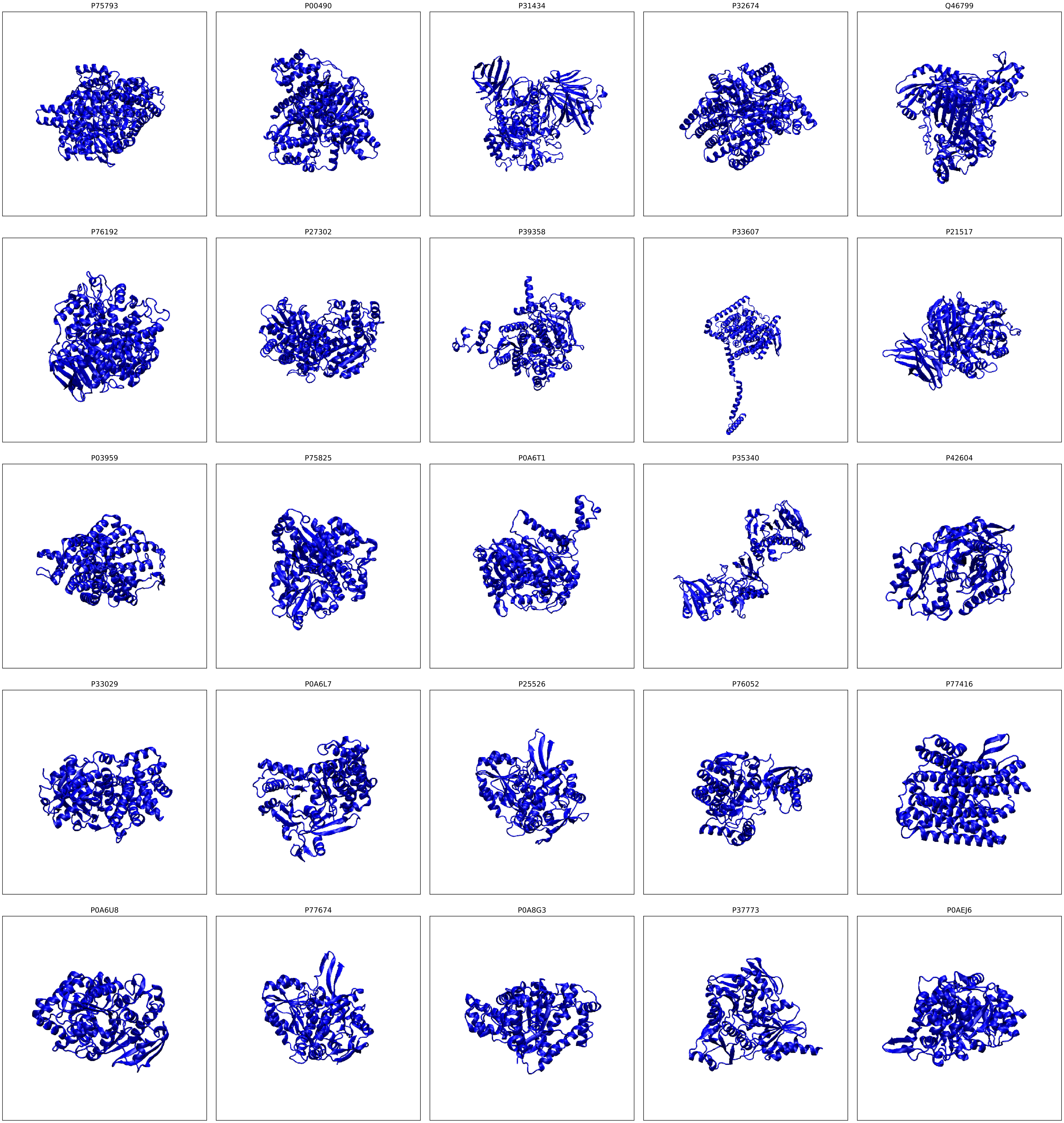

YEAST catalog top 25 entries

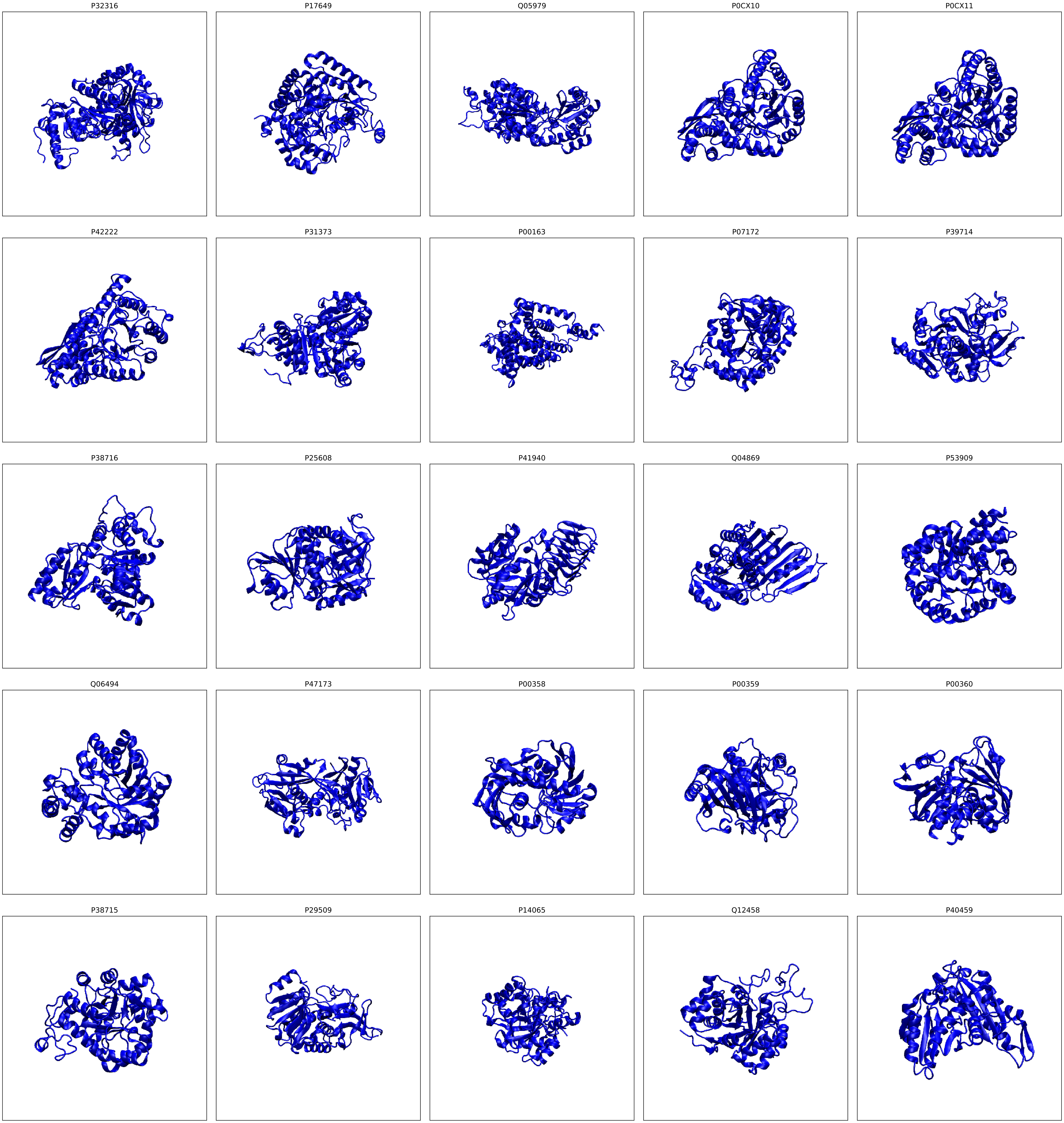

SCHPO catalog top 25 entries

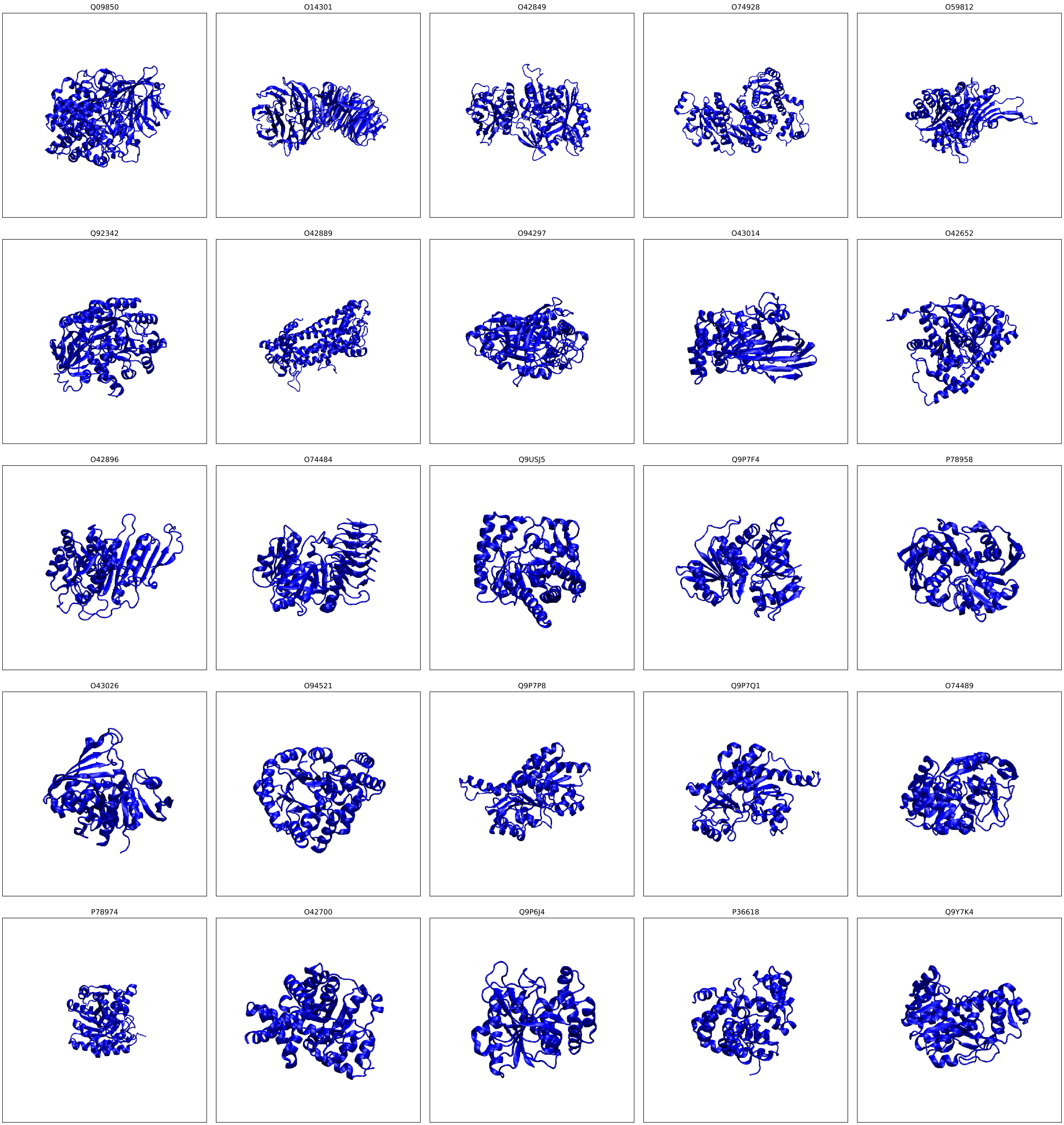

METJA catalog top 25 entries

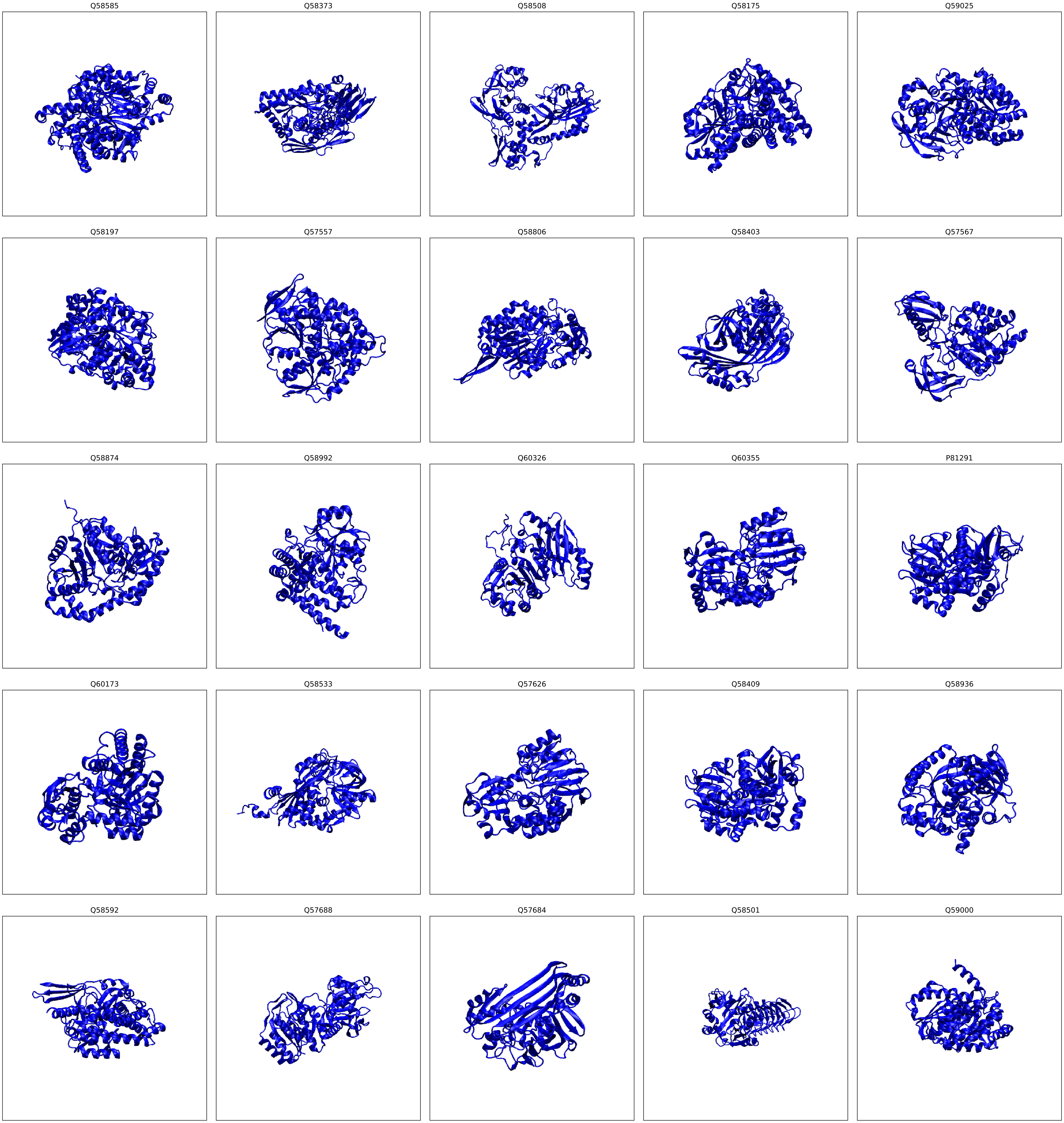

STAA8 catalog top 25 entries

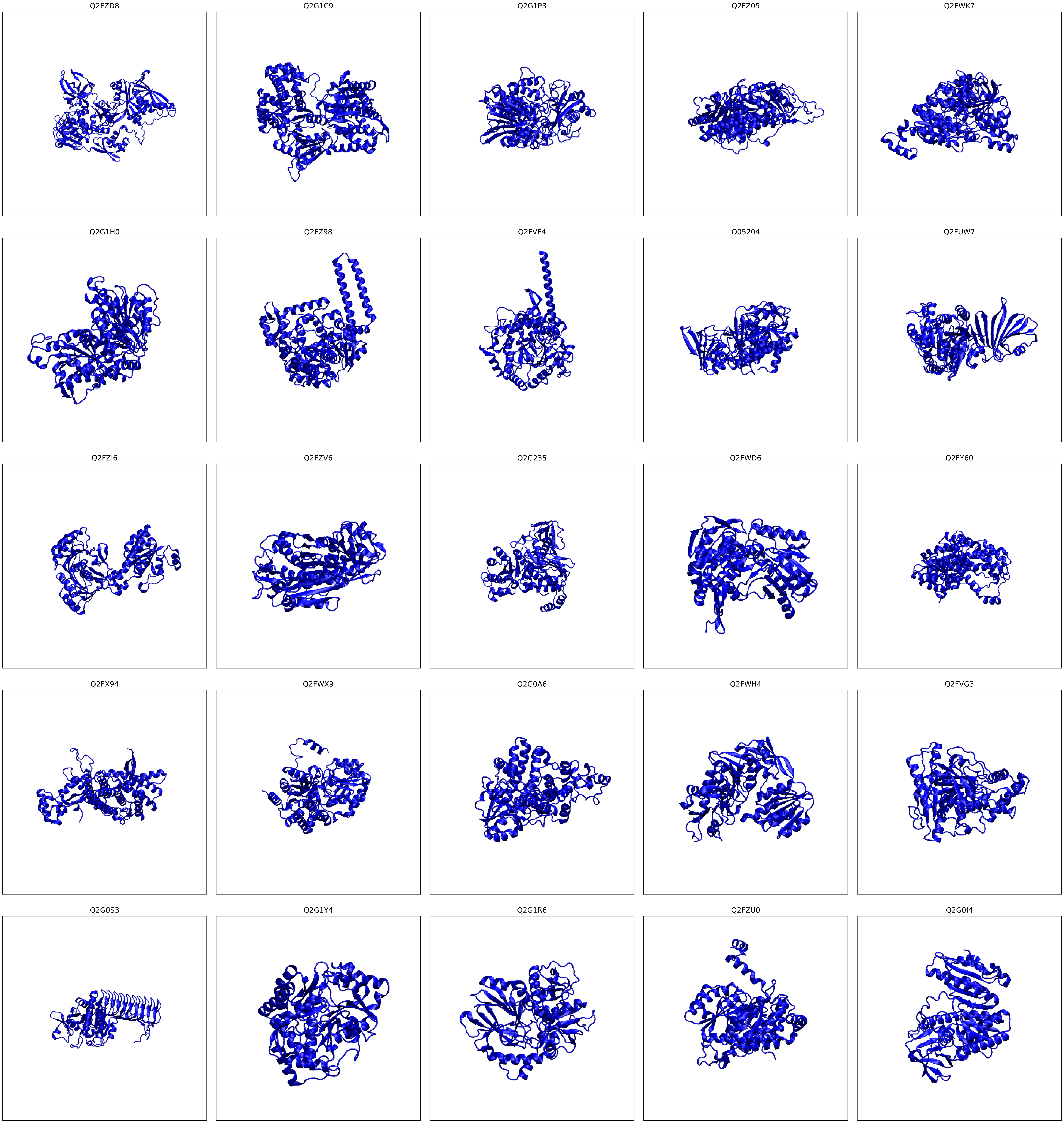

PLAF7 catalog top 25 entries

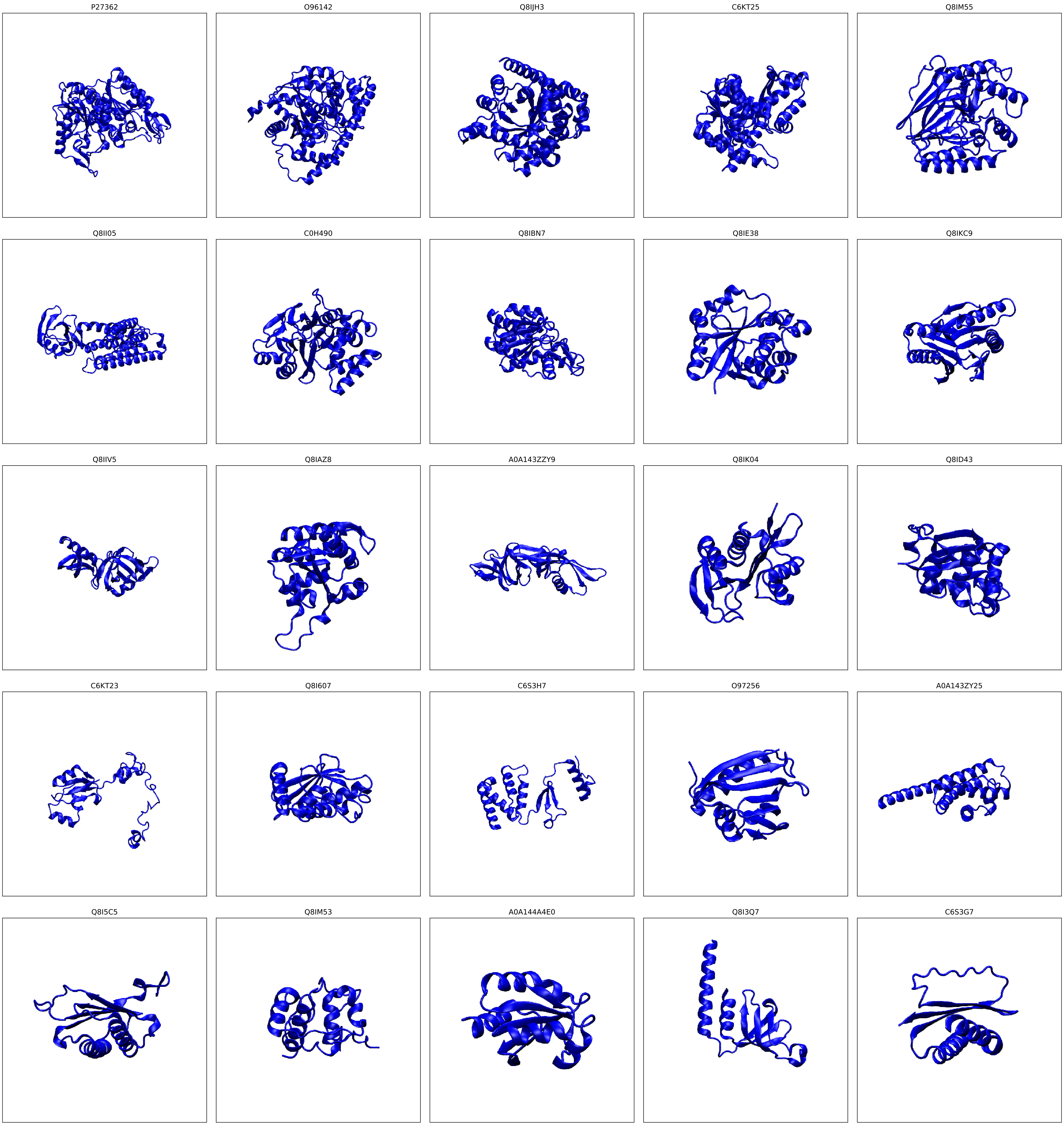

PSEAE catalog top 25 entries

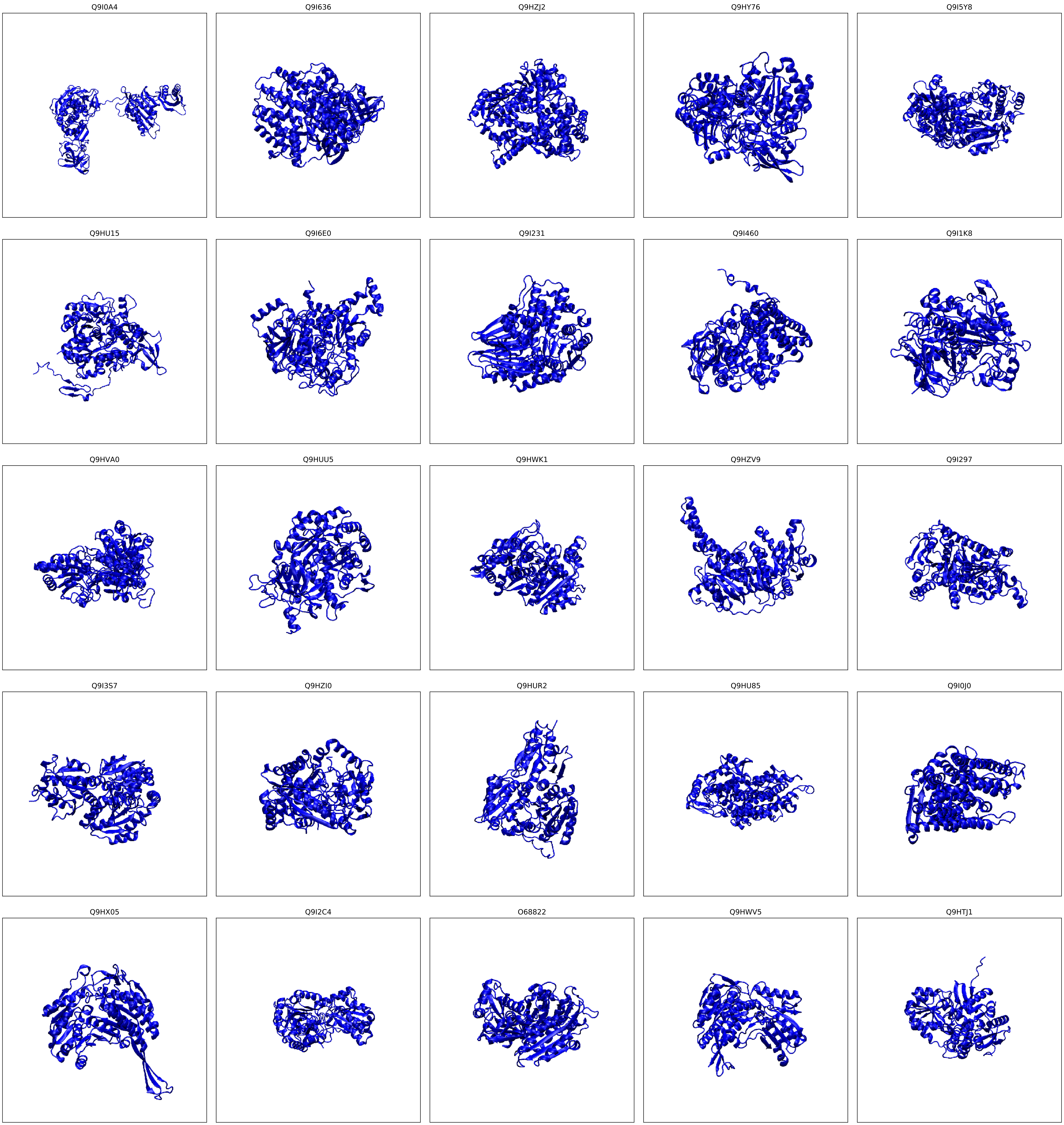

HELPY catalog top 25 entries

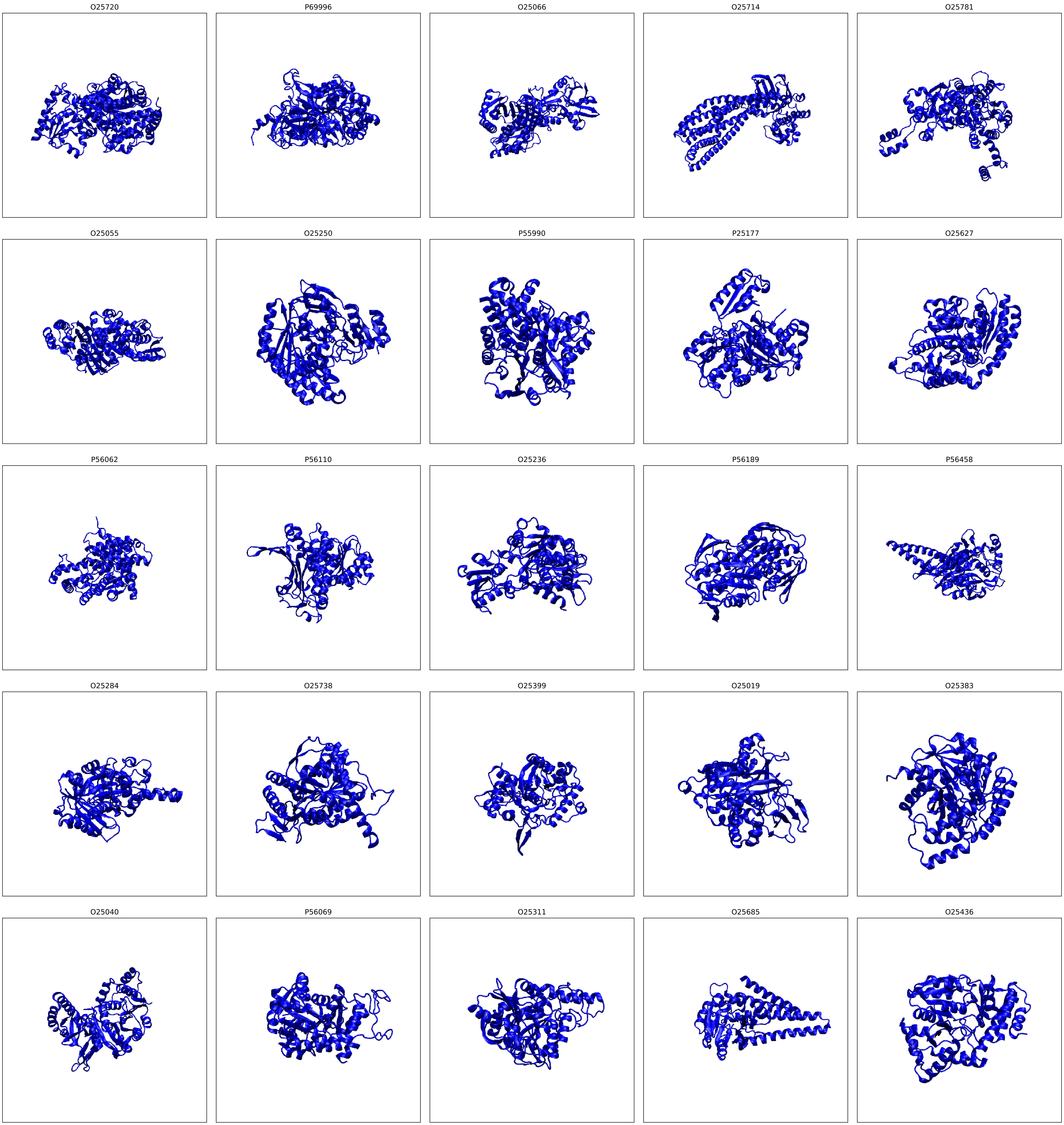

MYCTU catalog top 25 entries

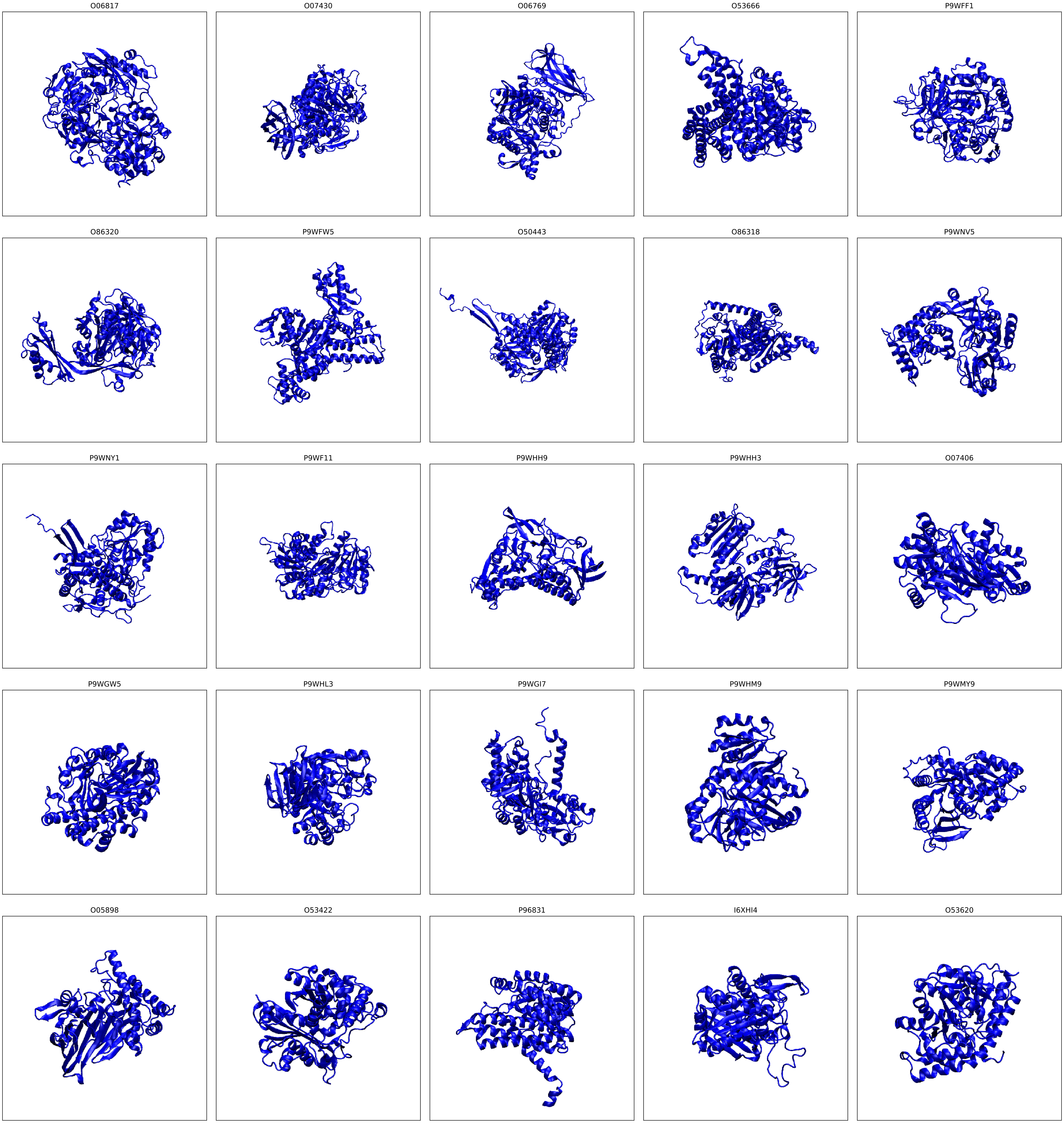

SHIDS catalog top 25 entries

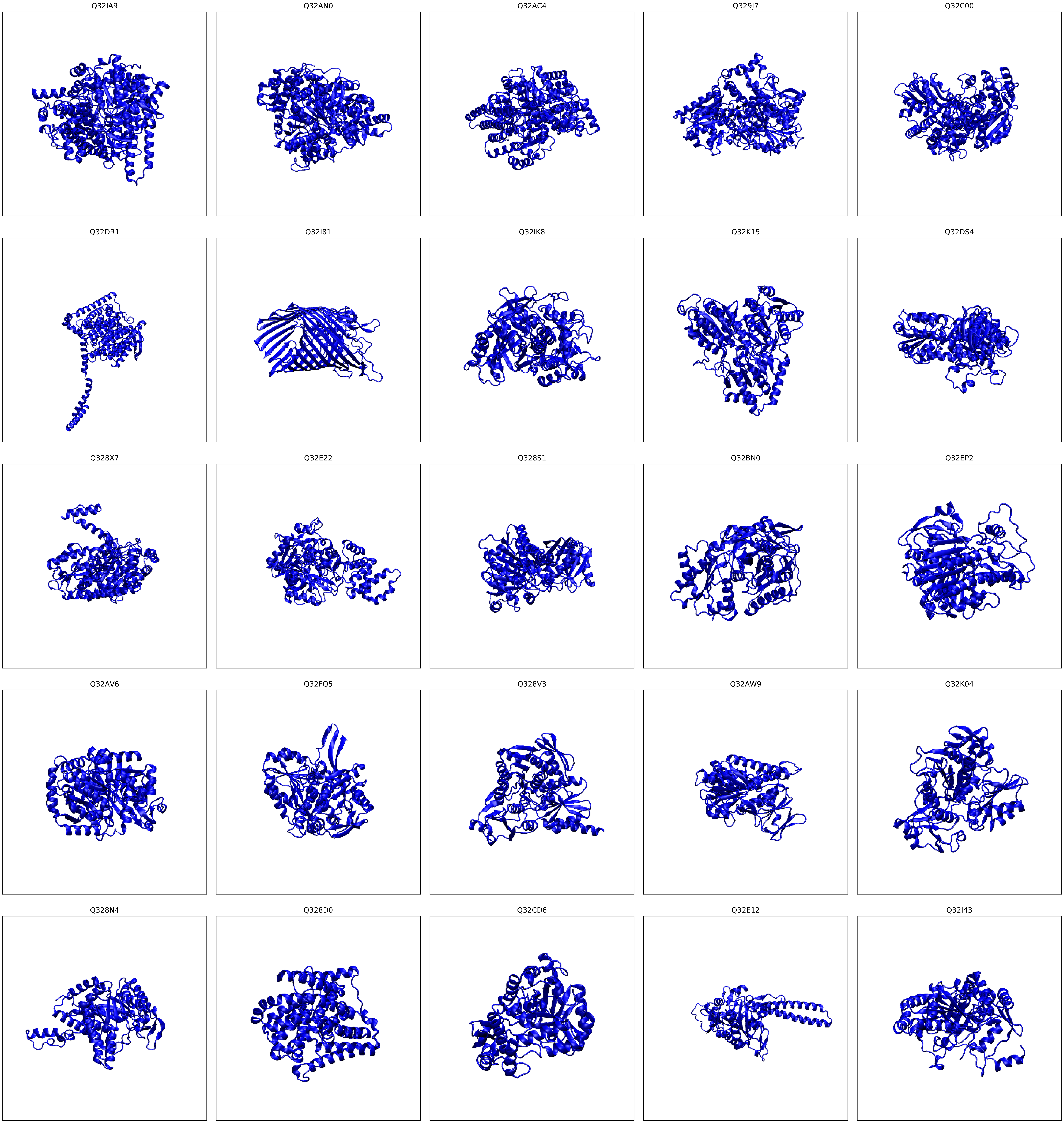

CAMJE catalog top 25 entries

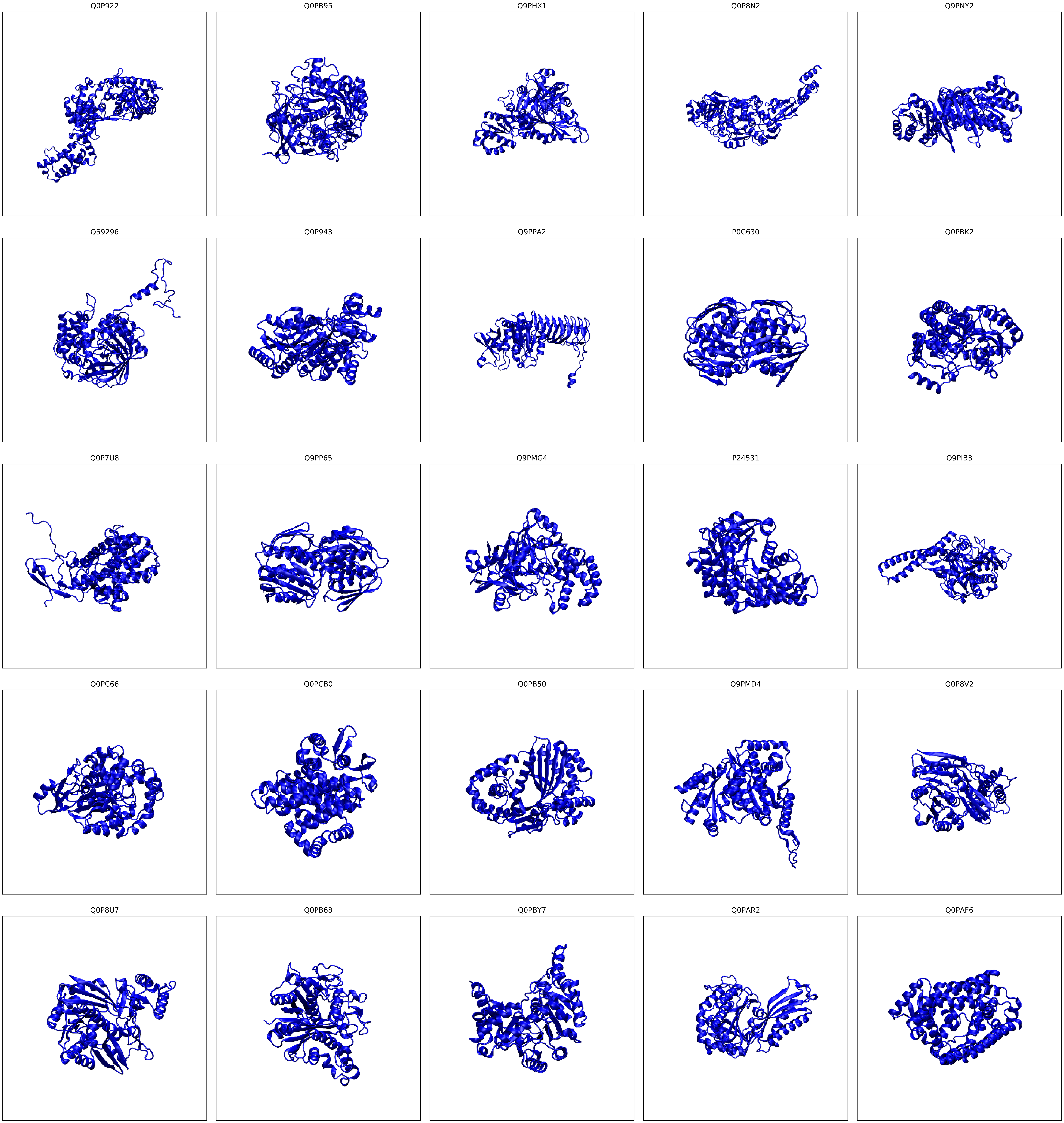

SALTY catalog top 25 entries

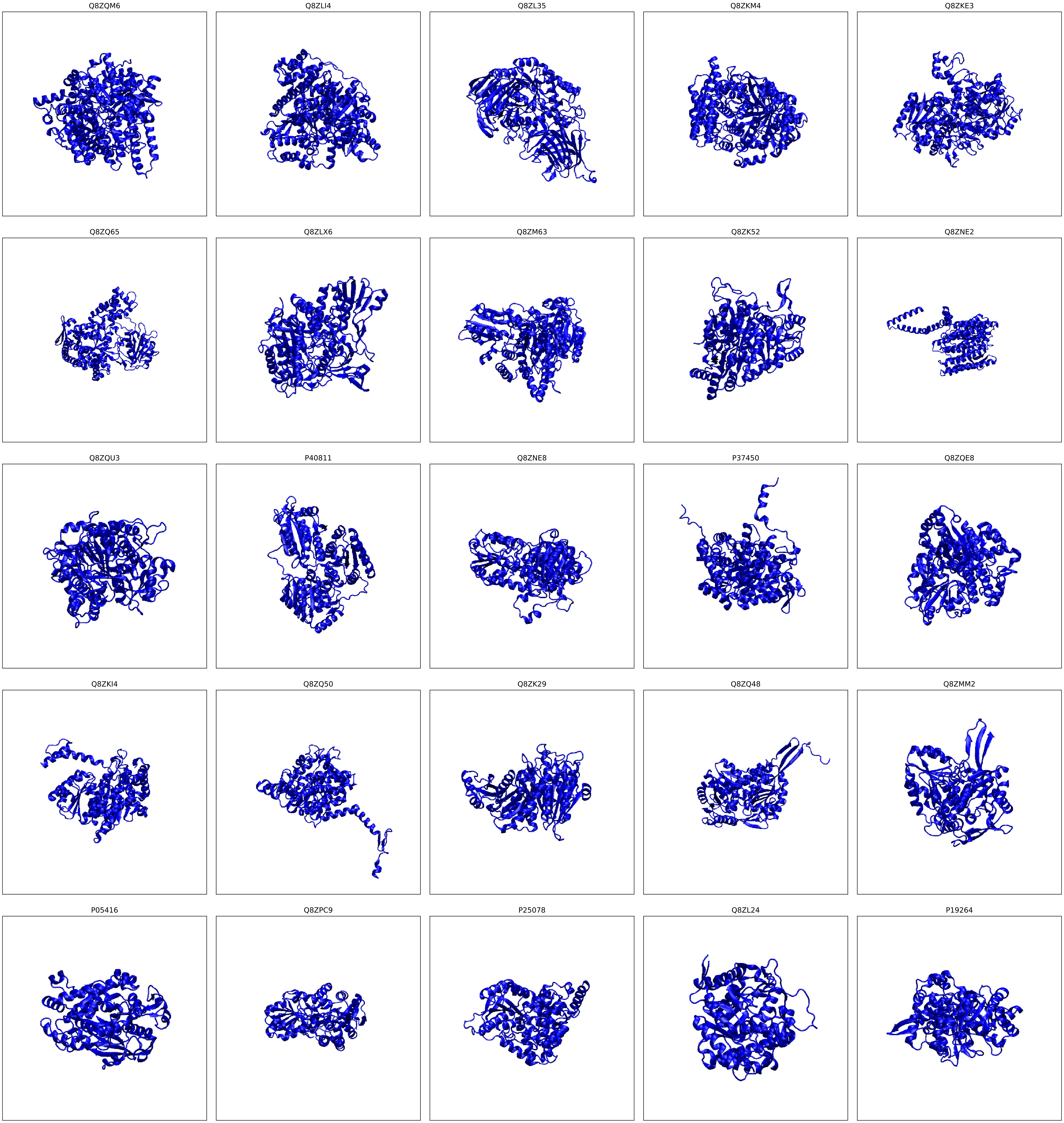

HAEIN catalog top 25 entries

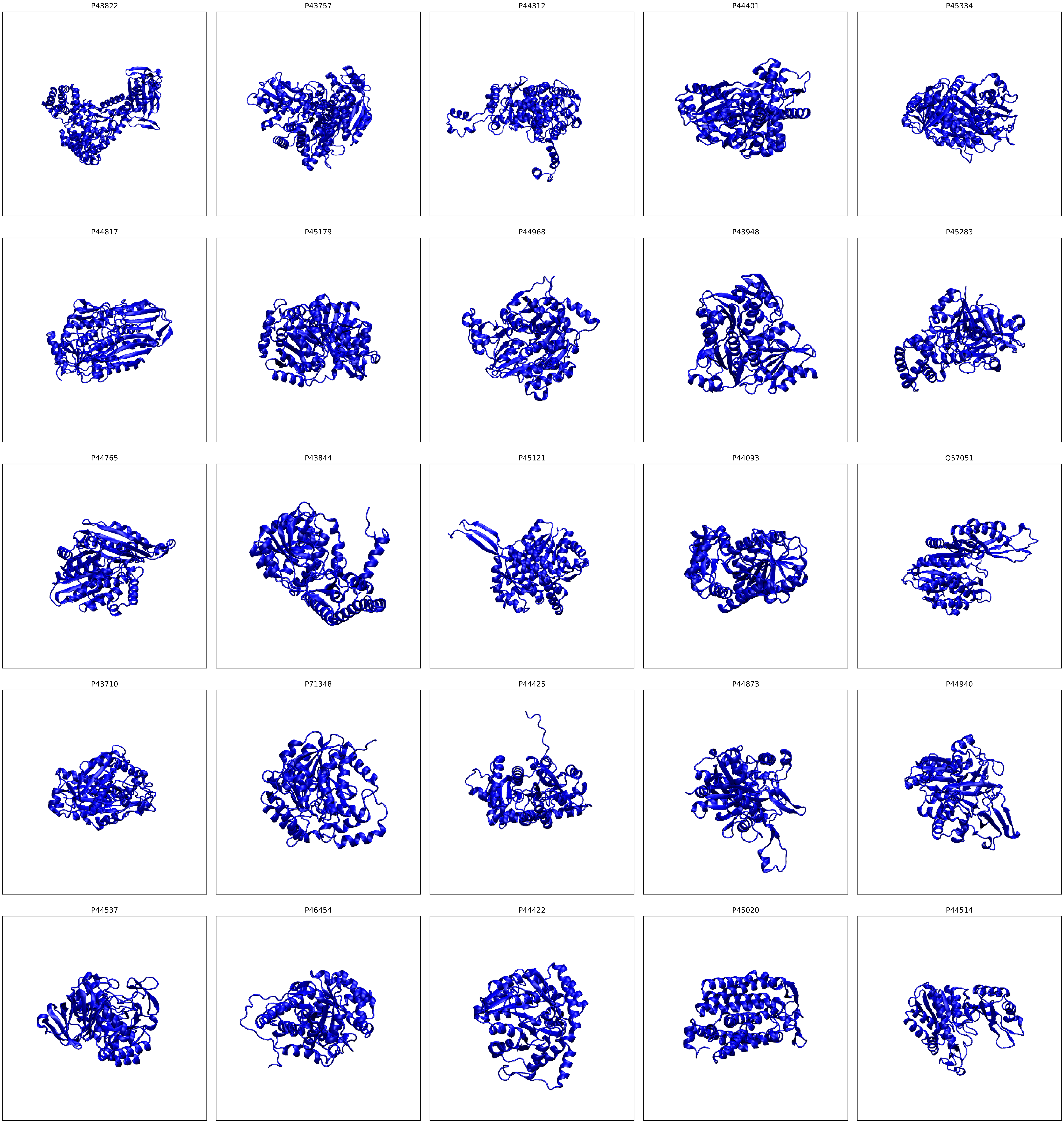

STRR6 catalog top 25 entries

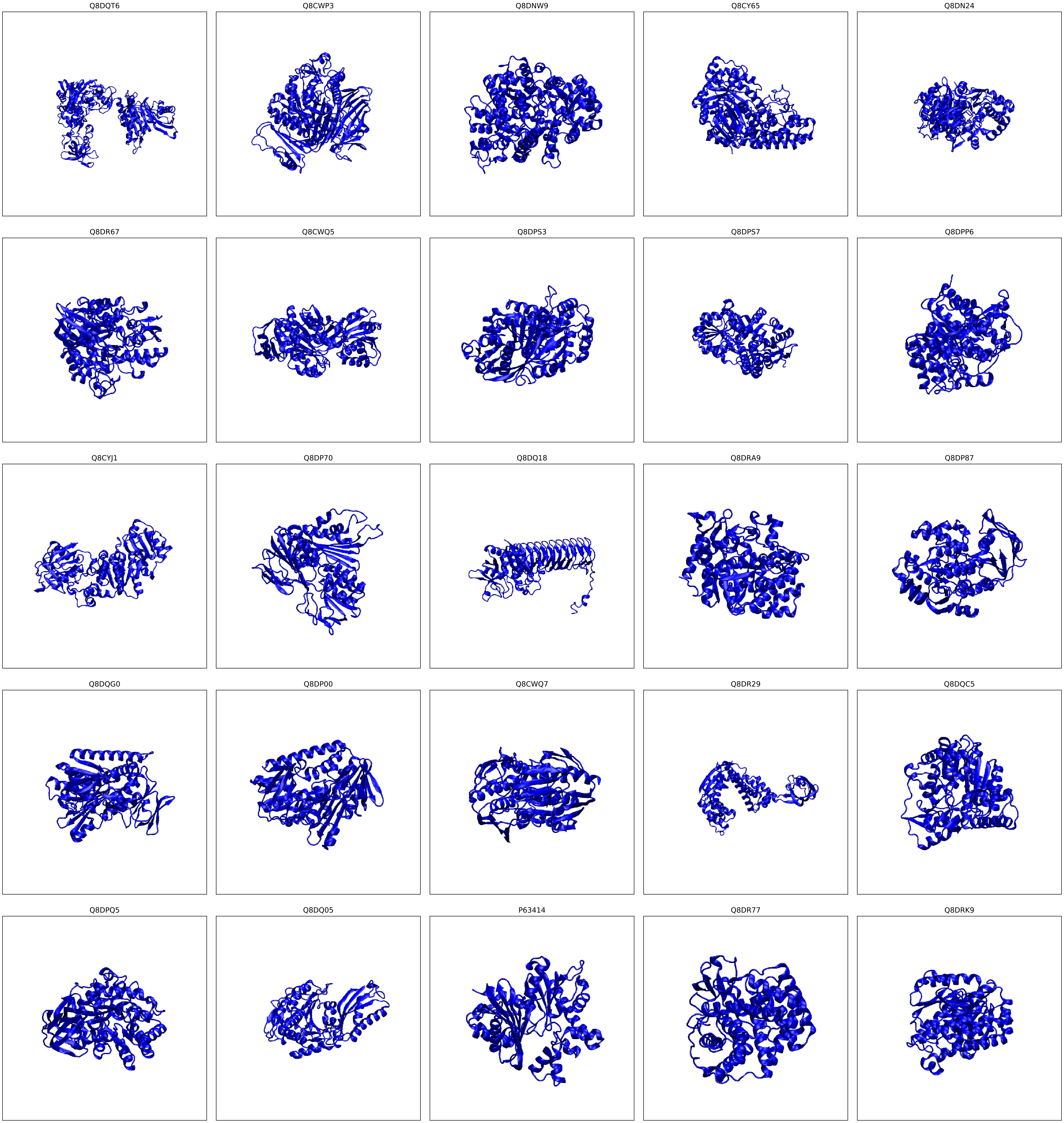

KLEPH catalog top 25 entries

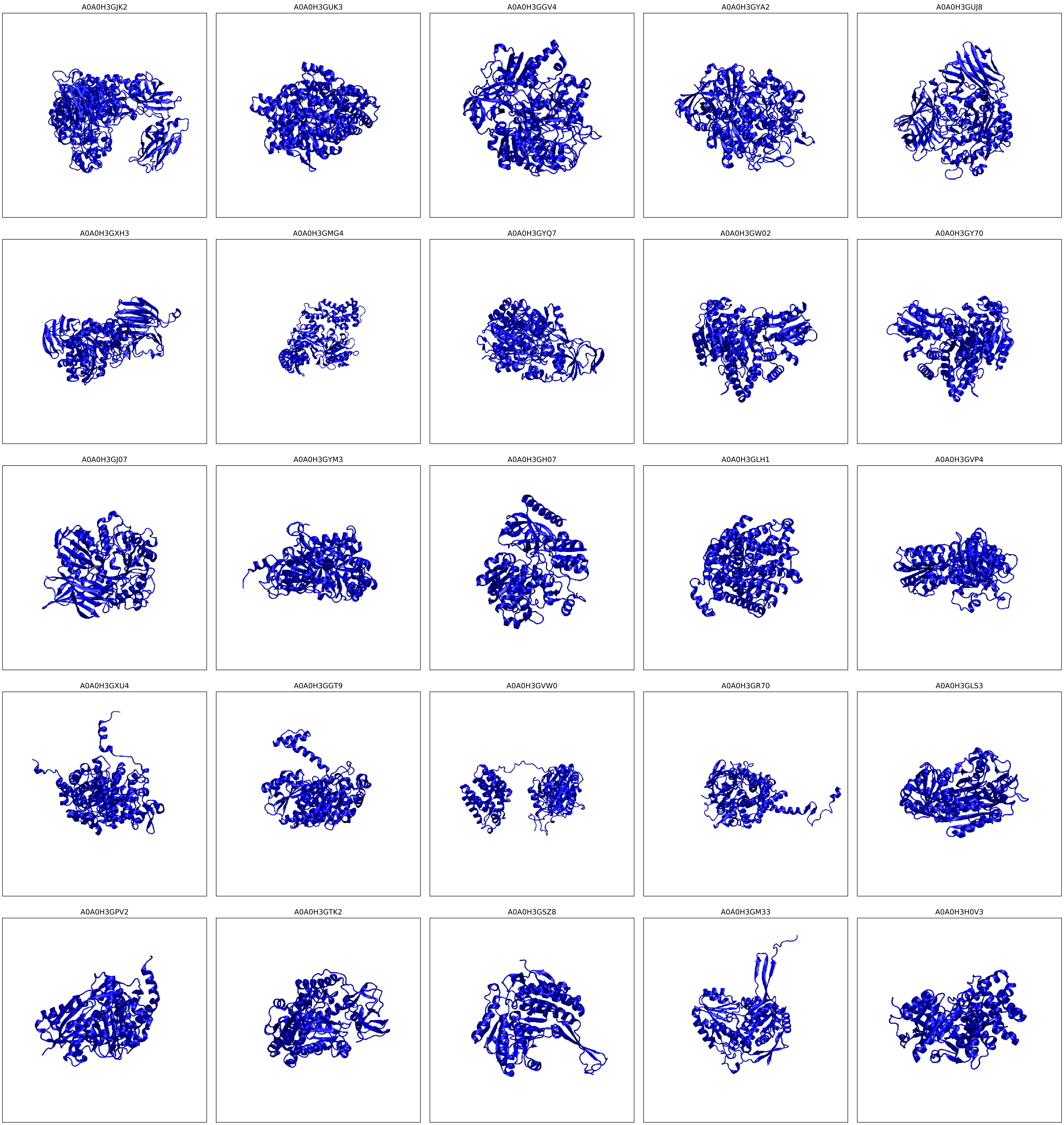

NEIG1 catalog top 25 entries

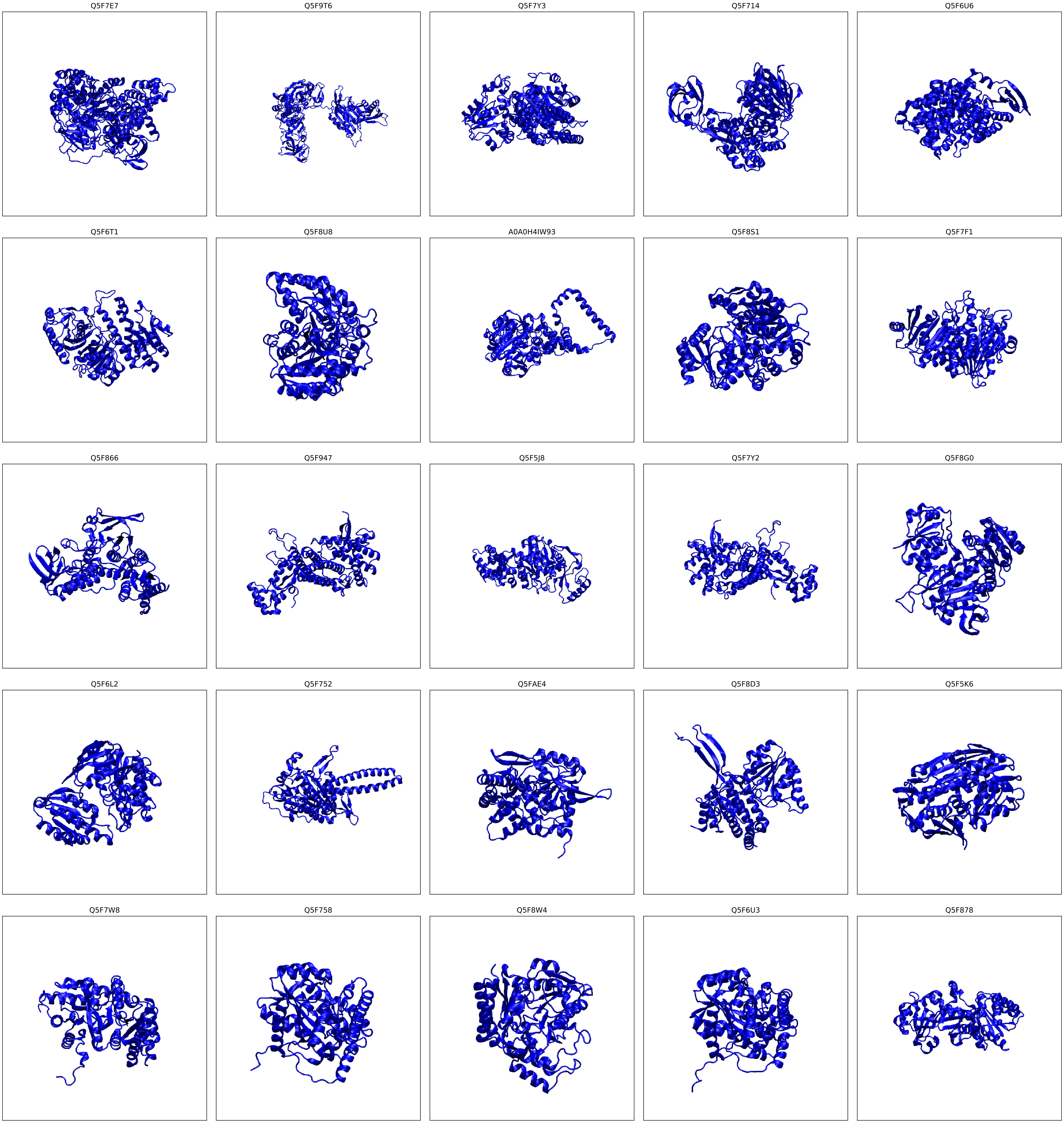

MYCLE catalog top 25 entries

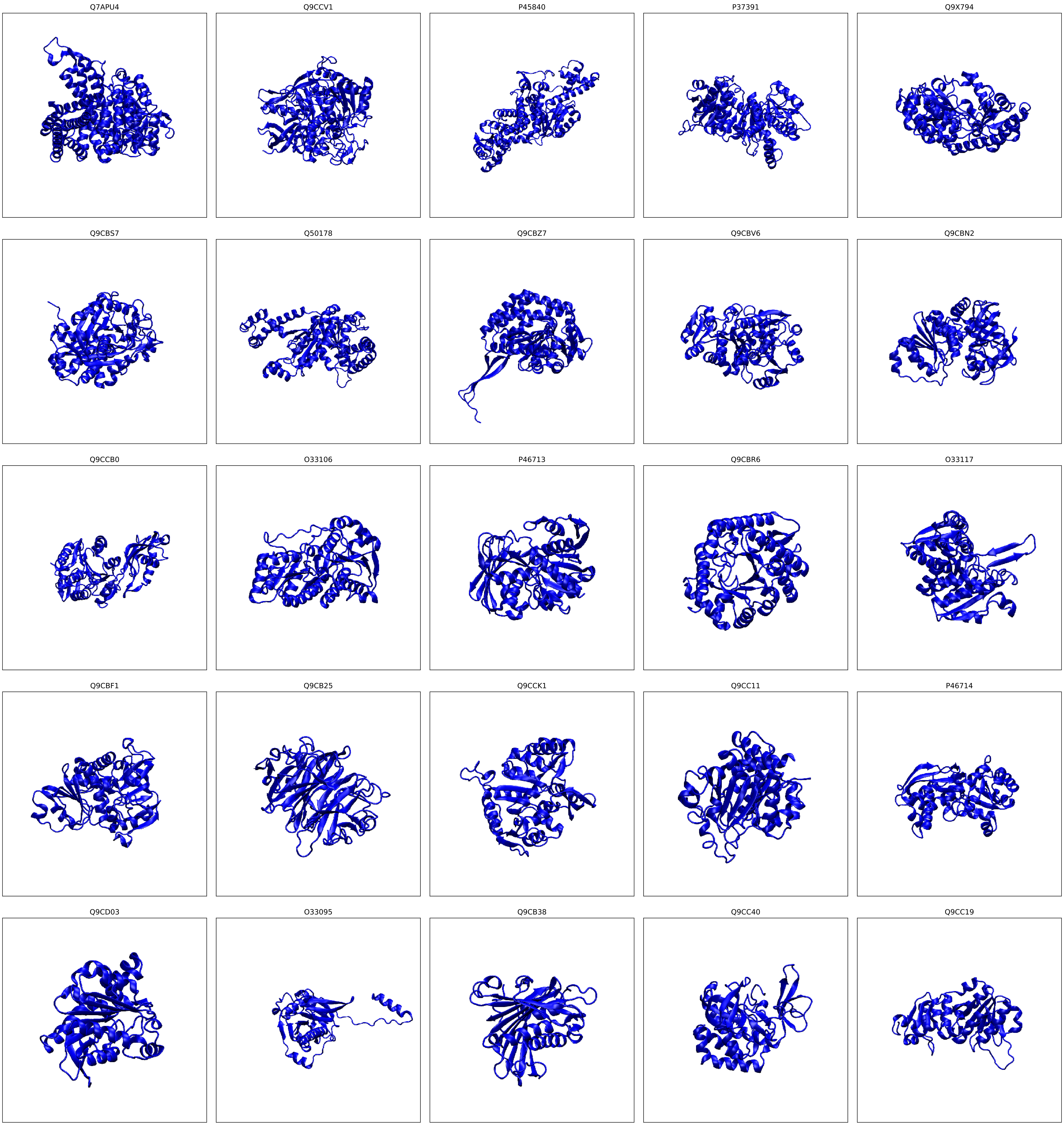

ENTFC catalog top 25 entries

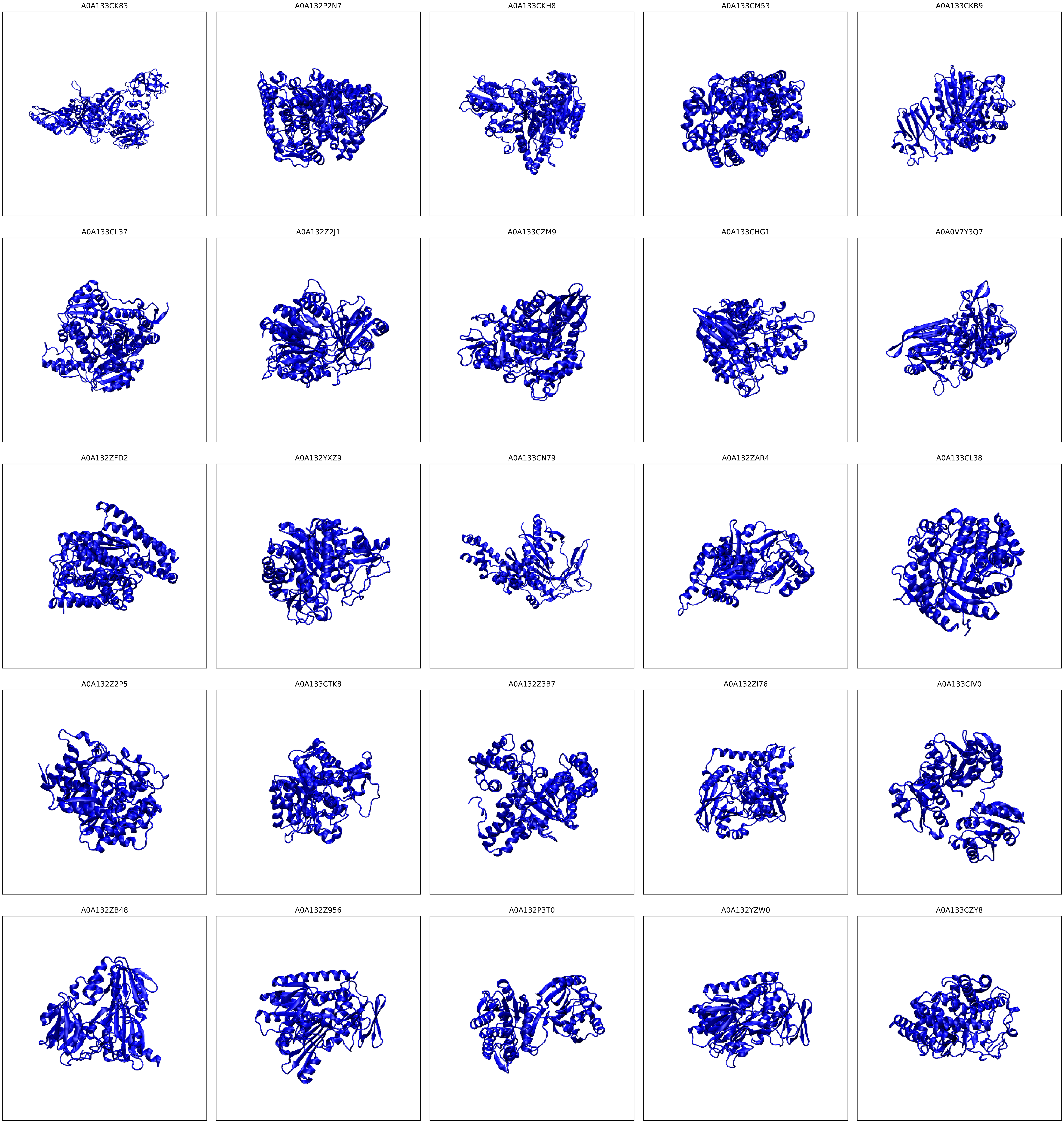

Supplement: gkac828_Supplemental_Files [file gkac828_supplemental_files.zip › Supplementary_Figure_1.pdf]
